# A prognostic risk score for development and spread of chronic pain

**DOI:** 10.1038/s41591-023-02430-4

**Published:** 2023-07-06

**Authors:** Christophe Tanguay-Sabourin, Matt Fillingim, Gianluca V. Guglietti, Azin Zare, Marc Parisien, Jax Norman, Hilary Sweatman, Ronrick Da-ano, Eveliina Heikkala, PREVENT-AD Research Group, John C. S. Breitner, Julien Menes, Judes Poirier, Jennifer Tremblay-Mercier, Jordi Perez, Jaro Karppinen, Sylvia Villeneuve, Scott J. Thompson, Marc O. Martel, Mathieu Roy, Luda Diatchenko, Etienne Vachon-Presseau

**Affiliations:** 1 Alan Edwards Centre for Research on Pain, McGill University, Montreal, Quebec Canada; 2 Faculty of Medicine, Université de Montréal, Montreal, Quebec Canada; 3 Department of Anesthesia, Faculty of Medicine and Health Sciences, McGill University, Montreal, Quebec Canada; 4 Faculty of Dental Medicine and Oral Health Sciences, McGill University, Montreal, Quebec Canada; 5 Department of Neurology and Neurosurgery, McGill University, Montreal, Quebec Canada; 6 Research Unit of Population Health, University of Oulu, Oulu, Finland; 7 Medical Research Center Oulu, University of Oulu and Oulu University Hospital, Oulu, Finland; 8 Alan Edwards Pain Management Unit, McGill University Health Centre, Montreal, Quebec Canada; 9 Research Unit of Health Sciences and Technology, University of Oulu, Oulu, Finland; 10 Rehabilitation Services of Southern Karelia Social and Health Care District, Lappeenranta, Finland; 11 Douglas Mental Health Institute Research Centre, McGill University, Montreal, Quebec Canada; 12 McConnell Brain Imaging Center, Montreal Neurological Institute, McGill University, Montreal, Quebec Canada; 13 Department of Anesthesiology, University of Minnesota, Minneapolis, MN USA; 14 Department of Psychology, McGill University, Montreal, Quebec Canada; 15 Centre for Studies on the Prevention of Alzheimer’s Disease, Douglas Mental Health University Institute, Montreal, Quebec Canada; 16 Department of Psychiatry, McGill University, Montreal, Quebec Canada

**Keywords:** Risk factors, Predictive markers

## Abstract

Chronic pain is a complex condition influenced by a combination of biological, psychological and social factors. Using data from the UK Biobank (*n* = 493,211), we showed that pain spreads from proximal to distal sites and developed a biopsychosocial model that predicted the number of coexisting pain sites. This data-driven model was used to identify a risk score that classified various chronic pain conditions (area under the curve (AUC) 0.70–0.88) and pain-related medical conditions (AUC 0.67–0.86). In longitudinal analyses, the risk score predicted the development of widespread chronic pain, the spreading of chronic pain across body sites and high-impact pain about 9 years later (AUC 0.68–0.78). Key risk factors included sleeplessness, feeling ‘fed-up’, tiredness, stressful life events and a body mass index >30. A simplified version of this score, named the risk of pain spreading, obtained similar predictive performance based on six simple questions with binarized answers. The risk of pain spreading was then validated in the Northern Finland Birth Cohort (*n* = 5,525) and the PREVENT-AD cohort (*n* = 178), obtaining comparable predictive performance. Our findings show that chronic pain conditions can be predicted from a common set of biopsychosocial factors, which can aid in tailoring research protocols, optimizing patient randomization in clinical trials and improving pain management.

## Abstract (web summary)

A risk score developed using biological, psychological and social factor data from the UK Biobank can predict different pain conditions, the risk of chronic pain spreading across body sites and the prognosis of chronic pain up to 9 years later.

## Main

Pain is the primary reason that individuals seek healthcare and is a leading cause of disability among working adults^1–3^. Unfortunately, the causes of chronic pain and its prognosis are often unknown, as tissue damage following injury is rarely an accurate predictor of clinical outcomes^4^. Instead, it is widely accepted that the interactions between biological, psychological and social factors play a greater role in determining chronic pain conditions and patients’ overall functioning^5^. This holistic framework, referred to as the biopsychosocial model for chronic pain^5^, can be challenging to define owing to the difficulties of simultaneously measuring and distinguishing multidimensional factors in large groups of patients living with pain. Access to large cohorts of participants with chronic pain has provided unprecedented opportunities to tackle these problems and better understand the determinants of chronic pain^6^.

Prognostic studies have shown that certain factors, such as maladaptive pain-coping strategies, somatization of pain and history of pain increase the likelihood of developing chronic back pain^4,7–9^. Additionally, factors including pain severity and duration^9–12^, fear of pain^13^ and pain catastrophizing^4,14^ have been linked to worsening back pain. Brain imaging and genetic studies also suggest that biological factors predispose individuals to chronic pain conditions^15^; however, these studies are often circular, as pain measurements or attitudes toward pain are used as pain predictors and most candidate brain-imaging markers have been identified in studies with small sample sizes, making them difficult to reproduce in larger and more diverse groups^16,17^. Furthermore, these previous prospective studies have rarely been validated in out-of-sample patients and the generalizability of the findings to new patients remains unknown^18,19^. A data-driven framework with clinical utility for predicting pain conditions is currently missing.

The Task Force for the Classification of Chronic Pain recommends classifying chronic pain conditions based on their etiology (for example, musculoskeletal pain), underlying pathophysiology (for example, neuropathic pain) or body site (for example, back pain)^20,21^. Despite differences between these conditions, evidence suggests that pain conditions overlap with one another^22^, share a common genetic risk profile^23,24^ and show similar alterations in the central nervous system^15,25,26^. Moreover, coexisting pain conditions, which over one-third of pain patients report experiencing, are associated with higher impact of pain, including lower quality of life and poorer response to treatment^22,27^. Thus, it is believed that different pain conditions share common risk factors and primary chronic pain is now recognized as a disease on its own rather than a symptom of another disease^28^.

**Fig. 1:**
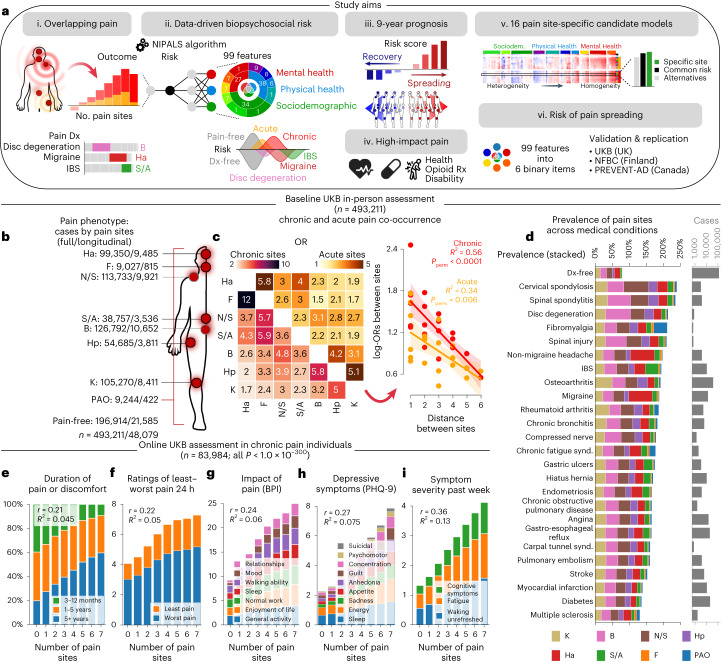
Phenotyping pain in the UK Biobank. **a**, Schematic showing the study workflow. IBS, irritable bowel syndrome; Dx, diagnosis; S/A, stomach or abdominal; B, back; Ha, headache; Rx, prescription; UKB, UK Biobank; NFBC, Northern Finland Birth Cohort; Sociodem., sociodemographic. **b**, Anatomical body map of pain sites and counts of pain cases (combined acute and chronic) for the full sample and for individuals with a follow-up visit 9 years later (in-person assessment). F, facial; N/S, neck or shoulder; Hp, hip; K, knee; PAO, pain all over. **c**, Odds ratios (ORs) of co-occurrence between pain sites (chronic on the left and acute on right) at baseline. The log-OR of co-occurring pain between two sites were negatively associated with their distances in chronic (*P*_perm_ < 0.0001) and acute pain (*P*_perm_ = 0.006, using 10,000 two-sided permutation (perm) tests). The 95% confidence interval (CI) estimated across 1,000 bootstrap samples is shown. **d**, The prevalence of pain is shown per body site among noncancer medical conditions commonly associated with chronic pain and the count of cases reported. **e**–**i**, In the online assessment pain questionnaire in chronic pain individuals, the number of coexisting pain sites (0 indicates no major sites) was associated (two-sided Pearson’s *r* correlations, all *P* < 1.0 × 10^−300^) with the duration or discomfort of pain (**e**), rating of the least and worst pain out of 10 in the last 24 h (**f**), interference of pain across seven dimensions (**g**), depressive symptom severity in last 2 weeks (**h**) and symptom severity during the last week (**i**). BPI, brief pain inventory; PHQ, patient health questionnaire.

Building on these ideas, we applied machine learning to the UK Biobank dataset to synthesize a wide range of multidimensional pain-agnostic features and develop a predictive model capable of classifying and forecasting different pain conditions and the spreading of pain across body sites. Our first hypothesis was that different chronic pain conditions are characterized by a common set of psychosocial factors that can be identified by studying the number of coexisting pain sites. The second hypothesis was that these risk factors can predict the development of various chronic pain conditions. The third hypothesis was that the differences between the observed pain and the predicted pain based on these risk factors will determine the spreading or recovery of chronic pain about 9 years later. We also conducted exploratory analyses to evaluate the following aspects of our model: its ability to predict high-impact pain, the specificity of candidate models trained on different body sites and the development of a simplified version of our model for generalizable use in research or clinical settings. Figure 1a illustrates the study workflow, highlighting each hypothesis (Fig. 1a(i–iii)) and exploratory analysis (Fig. 1a(iv–vi)).

## Results

This study was conducted using data from the UK Biobank (timeline shown in Supplementary Fig. 1). At their initial visit, participants were asked whether they experienced pain interfering with their usual activities in the last month at the following body sites: head, face, neck/shoulder, stomach/abdominal, back, hip and knee. The participants could also respond that they experienced pain all over the body (PAO) or none of the above (the latter were categorized as pain-free participants). Figure 1b shows the prevalence of pain in the full sample of participants (*n* = 493,211) and a subsample of participants who returned for a follow-up magnetic resonance imaging (MRI) visit (median time between visits of 9 years; *n* = 48,079). Participants reporting pain were then asked whether they had pain lasting for more than 3 months, which represents the cutoff for the pain to be considered chronic^20^. Pain experienced for less than 3 months was considered to be acute.

### Overlapping pain

In the UK Biobank, 44% of individuals experiencing chronic pain reported pain at more than one body site and the co-occurrence of pain was more frequent between proximal sites than distal sites (*R*^2^ = 0.56, *P*_perm_ < 0.0001; Fig. 1c). This pattern was also observed in acute pain conditions (*R*^2^ = 0.34, *P*_perm_ < 0.006). These results indicate that pain was not amplified uniformly across body sites in either acute or chronic pain. We then examined the prevalence of these pain conditions across a series of common self-reported clinical diagnoses. Here, pain conditions and other pain-related medical conditions were all characterized by overlapping pain conditions (Fig. 1d). For example, in the case of migraine, non-migraine headache or spinal spondylitis, the prevalence of pain at the head (migraine and non-migraine headache) or back (spinal spondylitis) sites were lower than the cumulative prevalence of pain at the remaining sites.

**Extended Data Fig. 1:**
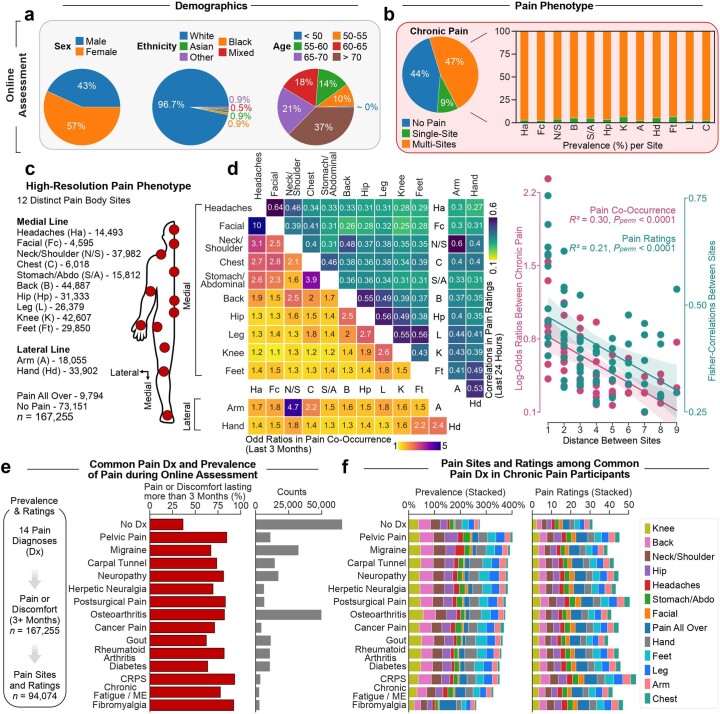
Online UK Biobank assessment of the experience of pain. **a**. Demographics of participants across sex, ethnicity and age. **b**. Pain reported in the past 3 months (chronic pain, > 3 months) for single and multi-site pain. **c**. High-resolution representation of anatomical body map sites and counts across a total of 13 sites: 10 along the medial line, 2 along the lateral line (shoulder to arm-hand) and 1 not localized (widespread). PAO, pain all over; Ha, headache; Fc, facial; N/S, neck or shoulder; C, chest; S/A, stomach or abdominal; B, back; Hp, hip; L, leg; K, knee; Ft, feet; A, arm; Hd, hand. **d**. Cross-sectional analysis of co-existing pain and pain ratings. Odds ratios (OR) of co-occurrence between sites in the past 3 months (left diagonal, yellow) and Pearson’s *r* correlations between pain ratings in the last 24 hours (right diagonal; green). Both the log- normalized OR of pain sites (*P*_perm_ < 0.0001) and fisher- normalized *r* correlations (*P*_perm_ < 0.0001, using 10,000 two-sided permutation tests) were negatively associated with their distance. 95% Confidence interval estimated across 1,000 bootstrap samples is shown. **e-f**. A total of 14 common chronic pain diagnoses were included. **e**. Counts of diagnoses across the entire online assessment and the prevalence of those reporting pain or discomfort in the past 3 months. No Dx includes those without any of the 14 diagnoses. **f**. Pain prevalence and mean pain ratings (10, as bad as you can imagine) across each diagnosis stacked across body sites.

The role of coexisting pain conditions was then examined using an online pain assessment of 84,030 individuals reporting chronic pain, excluding pain all over the body. The number of pain sites reported at the time of the online assessment (November 2019 to 2020) showed a monotonic increase with pain duration (*r* = 0.21; Fig. 1e), pain intensity (*r* = 0.22; Fig. 1f), impact of pain (*r* = 0.24; Fig. 1g), depressive symptoms (*r* = 0.27; Fig. 1h) and symptom severity (*r* = 0.36, Fig. 1i; all *P* < 1.0 × 10^−300^). The use of higher-resolution anatomical body sites in the online questionnaire further confirmed the spatial co-occurrence (*R*^2^ = 0.30, *P*_perm_ < 0.0001) and interdependence in pain ratings across sites in chronic pain (*R*^2^ = 0.21*, P*_perm_ < 0.0001; Extended Data Fig. 1). Here, diagnosed clinical conditions such as pelvic pain or carpal tunnel syndrome were characterized by coexisting pain at other body sites. These results show that the number of coexisting pain sites is an important phenotype characterizing different chronic pain conditions and reflecting the severity and impact of these pain conditions. We conclude that the number of coexisting pain sites is an effective target to train a predictive model for several different pain conditions.

### A data-driven biopsychosocial risk score for pain

**Extended Data Fig. 2:**
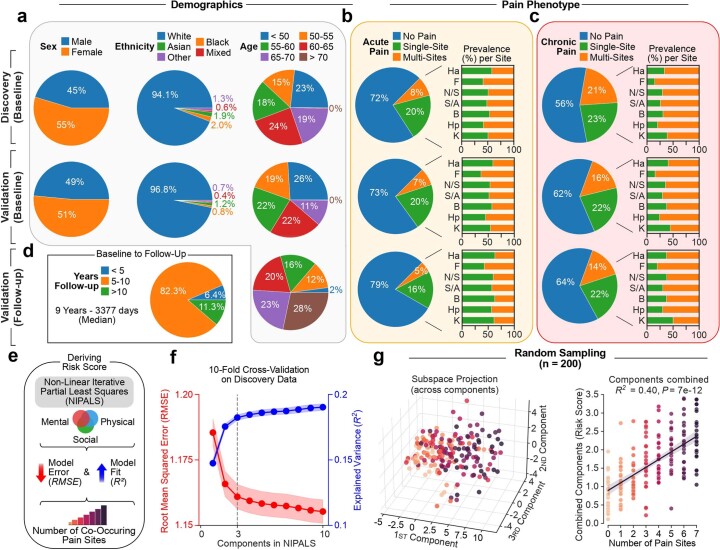
Discovery and Validation data for the risk score development. Pie charts displaying **a**. demographics, **b**. acute (≤3 months) and **c**. chronic (>3 months) pain phenotypes for the discovery data the model is trained on and the validation data the model is tested on, at baseline and follow-up. **d**. Years between baseline and follow-up visit in the validation data (9 years median). **e**. Schematic on using NIPALS to predict co-existing pain from biopsychosocial features. **f**. Model specification based on tenfold cross-validation by minimizing the root mean squared error (RMSE) and maximizing the explained variance (R^2^) average across tenfolds. Following the scree plot (elbow rule) criterion and to minimize overfitting, 3 components were selected**. g**. Random stratified sampling of 200 participants projected across the 3 components separately and combined as our risk score.

We used machine learning algorithms on 99 pain-agnostic features, including physical, psychological, demographic and sociological factors, to create a risk score that predicts the number of pain sites. To this end, the UK Biobank dataset available at the baseline visit (in-person assessment) was divided into a training set (*n* = 445,132) for discovery and a testing set composed of out-of-sample participants for whom longitudinal data were available (*n* = 48,079). We applied a nonlinear iterative partial least square (NIPALS)^29^ regression algorithm on the 99 features to predict the number of coexisting pain sites (combining acute and chronic) in the discovery set. The algorithm was trained using tenfold cross-validation to estimate the model fit and identify the optimal number of components (Extended Data Fig. 2). The trained model was then applied to the participants of the testing set.

**Fig. 2:**
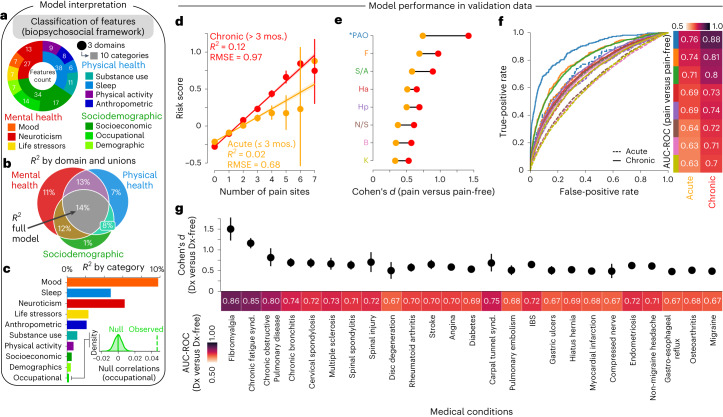
A multivariate model classifying and predicting different pain conditions. **a**, Classification of 99 clinical features grouped in three domains and ten categories. **b**, Venn diagram and bar graph show the model’s explained variance (*R*^2^) (ordered based on discovery results) in the number of pain sites across the three domains. **c**, The variance explained is shown for the ten categories and the category contributing the least was compared to a null model generated from 10,000 permutations**. d**, The model performance is shown in the testing set (validation data) using explained variance and root mean squared error (RMSE) for acute and chronic pain conditions separately (*n*_chronic_ = 17,948; *n*_acute_ = 13,117). Mean estimated across number of sites ± s.e.m. are shown. **e**, Cohen’s *d* effect sizes in the risk score for each pain site (acute in orange and chronic in red) compared to pain-free individuals. **f**, The diagnostic ability of our model to classify acute and chronic pain conditions is displayed using the AUC-ROC. **g**, The diagnostic ability of our model to classify the selected medical conditions is displayed using Cohen’s *d* and measured with AUC-ROC (selected Dx compared to Dx-free individuals). The 95% CI estimated across 1,000 bootstrap samples is shown. *PAO was excluded from model training in the discovery set. Dx, diagnoses.

**Extended Data Fig. 3:**
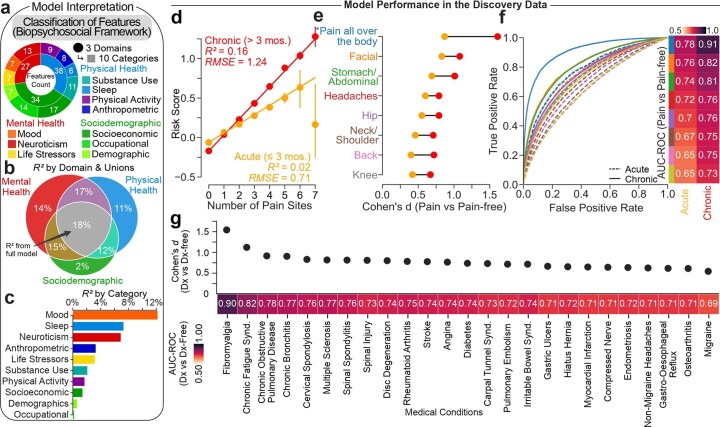
Model interpretation and performance in the discovery data. **a–g. Identical analyses conducted in the original discovery from which the model was derived (see** Fig. 2**). a**. Classification of 99 clinical features grouped in three domains and ten categories. **b**. Venn diagram and bar graph show the model’s explained variance (ordered based on discovery results) in the number of pain sites across the three domains **c**. The variance explained is shown for the ten categories **d**. The model performance is shown in the training set (*that is*, discovery data) using explained variance (R^2^) and Root Mean Squared Error (RMSE) for acute and chronic pain conditions separately (n _Chronic_ = 196,706, n _Acute_ = 126,313). Mean estimated across number of sites +/-standard errors are shown. **e**. Cohen’s *d* effect sizes in the risk score for each pain site (acute in orange and chronic in red) compared to pain-free individuals. **f**. The diagnostic ability of our model to classify acute and chronic pain conditions is displayed using AUC-ROC. AUC, area under the curve; ROC, receiver operating characteristic. **g**. The diagnostic ability of our model to classify the selected medical conditions is displayed using Cohen’s *d* and measured with AUC-ROC (selected Dx compared to Dx-free individuals). Error bars estimated from 10,000 bootstrap resampling are shown. *Pain all over the body was excluded from model training in the discovery set. Dx, diagnoses.

**Extended Data Fig. 4:**
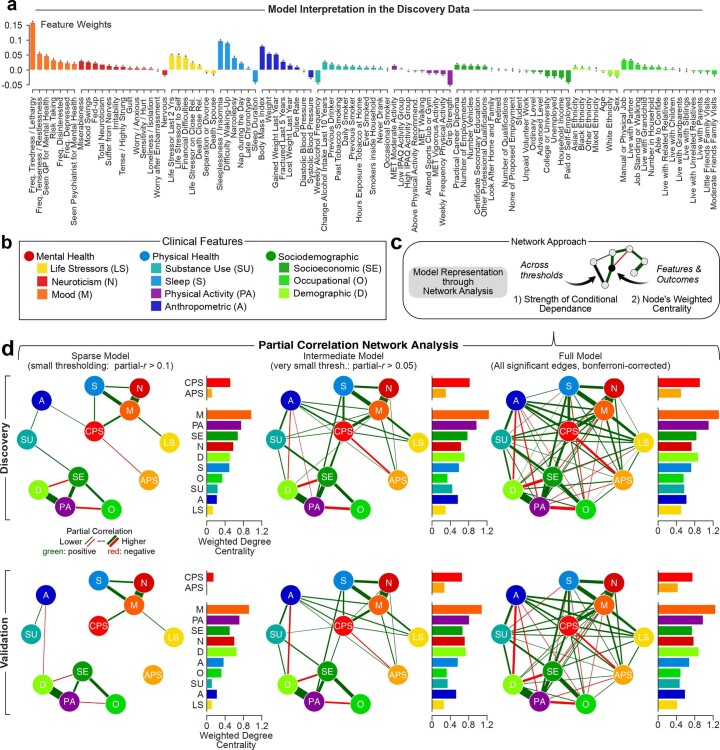
Model features and model integration through network analysis. **a**. Model feature weights projected to the risk score. Error distributions estimated across 1,000 bootstrap samples are shown. **b**. Schematic of a network approach to integrate the risk model’s categories. Edges and nodes were evaluated using strength of partial correlation and weighted node centrality. LS, life stressors; N, neuroticism; M, mood; SU, substance use; S, sleep; PA, physical activity; A, anthropometric; SE, socioeconomic; O, occupational; D, demographic. **c**. Schematic of the network analysis approach examining the centrality of each category and its connections across thresholds. **d**. Partial correlation network analyses across three levels: sparse (absolute partial correlation above 0.1), intermediate (above 0.05) and full (all edges) across the discovery data (upper row) and validation data (lower row).

The 99 features were organized into ten categories and three domains to improve the interpretability of the model (Fig. 2a). The model explained a total of 14% of the variance in the number of coexisting pain sites in the validation set (Fig. 2b), with the most explained variance coming from mood (12%), neuroticism (7%) and sleep (5%), whereas demographics and occupational measures explained the least variance (<1%; Fig. 2c). These results were consistent with those obtained in the discovery set (*R*^2^ of 12, 7 and 7%, respectively; Extended Data Fig. 3). A detailed list of features and their respective weights in the model is presented in Extended Data Fig. 4. Features in particular with positive weights included tiredness, insomnia and body mass index (BMI) and notable features with negative weights included grip strength, employment status and frequency of alcohol intake. Partial correlations were used to construct networks showing the respective contribution of each category for acute and chronic pain conditions, based on the strength of their conditional associations after controlling for other categories (Extended Data Fig. 4). Networks constructed at different densities consistently show that chronic pain (but not acute pain) was simultaneously associated with various categories (weighted centrality ranging from 0.15–0.60 for the chronic pain node and 0.0–0.32 for the acute pain node), highlighting the multifactorial nature of the model used to predict pain.

The model’s output provided a single prediction for the number of pain sites, for each participant, based on their score on the 99 features. This output, referred to as the risk score for pain, was used to predict the number of pain sites and classify each pain condition separately (Fig. 2d). The risk score for pain showed good to excellent performance for classifying participants with chronic pain conditions from pain-free participants at each body site, as shown by their effect sizes (Cohen’s *d* = 0.53–1.42; Fig. 2e) and diagnostic capacities (AUC 0.70–0.88, Fig. 2f). Although the model was trained on acute and chronic pain, the risk score for pain better predicted and classified chronic pain conditions than acute pain conditions (Cohen’s *d* = 0.33–0.74; AUC 0.63–0.76). Finally, the risk score for pain also showed good performance for classifying a broad range of medical conditions (Cohen’s *d* = 0.48–1.50; AUC 0.67–0.86; Fig. 2g).

### Recovery and spreading of chronic pain: 9-year prognosis

**Fig. 3:**
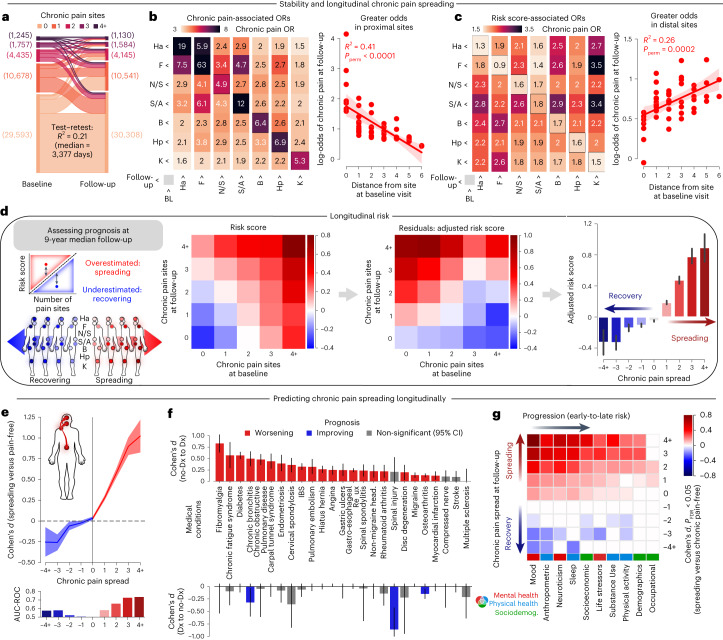
Forecasting the spreading and recovery of chronic pain. **a**, Test–retest variance explained (*R*^2^) in the number of chronic pain sites (4+ including PAO) between baseline and the follow-up visit. **b**, Odds of reporting chronic pain sites at baseline and the follow-up visit depended on the distance on the body map (*P*_perm_ < 0.0001). **c**, Our risk score, however, increased the odds of reporting pain at distal sites (*P*_perm_ = 0.0002, using 10,000 two-sided permutation tests). The 95% CI estimated across 1,000 bootstrap samples is shown. **d**, The matrices display the risk score depending on the changes in the number of chronic pain sites before (left matrix) and after (right matrix) adjusting linearly and exponentially for the number of chronic pain sites initially reported at baseline, age and years of follow-up. A negative-adjusted risk score was associated with recovery and a positive-adjusted risk score was associated with spreading of chronic pain. Means and s.e.m. are shown. **e**, The diagnostic capacities of our adjusted risk score for recovering and spreading was tested using Cohen’s *d* effect size (presented as mean ± s.e.m. estimated from 10,000 bootstrap samples) and AUC-ROC discrimination when compared to chronic pain-free participants. **f**, The same approach was conducted for diagnoses of medical conditions using Cohen’s *d* effect sizes (presented as mean ± s.e.m. estimated from 10,000 bootstrap samples). **g**, The order of progression between the pain determinants was determined using Cohen’s *d* in each category after controlling for multiple comparison. The factors are ordered depending on their importance in spreading and recovery. Early factors showed significant differences between small changes in chronic pain (for example, pain +1 or −1 site), whereas late factors only showed differences between large changes in chronic pain. FDR, false discovery rate.

We used the longitudinal dataset (the individuals from the test set that underwent a follow-up in-person assessment) to test whether the risk score for pain measured at baseline predicted changes in the number of chronic pain sites at the follow-up visit about 9 years later. The stability and individual changes in the number of pain sites between the two visits are displayed in Fig. 3a. The matrix in Fig. 3b shows that chronic pain at baseline was associated with higher odds of experiencing chronic pain at the same site or at a proximal site about 9 years later (*R*^2^ = 0.41, *P*_perm_ < 0.0001). Moreover, individuals with high risk scores for pain were more likely to report new pain at distal sites (*R*^2^ = 0.26, *P*_perm_ = 0.0002; Fig. 3c). This suggests that while baseline chronic pain presents a risk for the spreading of pain to proximal sites, a higher risk score for pain instead impacts the spreading of pain to distal sites, where pain does not normally propagate. As hypothesized, an elevated risk score for pain adjusted for the number of pain sites at baseline predisposed individuals to the pain outcomes measured at the follow-up visits; participants with negative scores recovered from their pain, whereas participants with positive scores progressed toward spreading of their pain (Fig. 3d). Thus, our adjusted risk score showed strong effect sizes, obtained good performance for predicting chronic pain spreading across multiple new pain sites at the follow-up visit (AUC 0.73 for 4+ sites; Fig. 3e) and predicted the prognosis of pain-related medical conditions in the longitudinal data (Cohen’s *d* = 0.25–0.83; Fig. 3f).

We next performed a tentative temporal ordering of individual risk factors by ranking the ten categories on the basis of their effect sizes to identify key risk factors that may indicate the onset or progression of chronic pain conditions. This procedure allowed us to unpack the sequence of the risk factors organized by categories, from early prodromal features to late features, predicting the progression of the spreading or recovering of chronic pain across body sites (Fig. 3g). The results show that mood was the earliest contributor to pain spreading, suggesting that mood-related factors may be early warning signs contributing to the development of chronic pain. On the other hand, occupation ranked last, suggesting that factors related to a job or career may not have as much impact on the development or progression of chronic pain spreading as other factors such as mood, anthropometric measurements, neuroticism or sleep. By establishing a tentative temporal ordering of these risk factors, we were able to better define the cascade of factors predicting chronic pain spreading, which could help develop more targeted interventions to prevent or manage pain conditions.

### High-impact pain

**Fig. 4:**
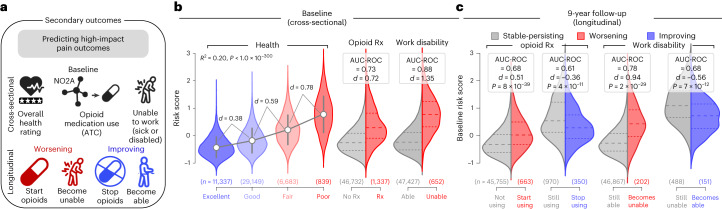
Predicting secondary outcomes associated with high-impact pain. **a**, Schematic of secondary outcomes examined: health ratings, opioid medications and disability and/or sickness. **b**, Cross-sectional performance of the risk score on secondary outcomes. Cohen’s *d* effect sizes and explained variance (*R*^2^, on the left with *P* estimated using a two-sided Pearson’s *r* correlations) were used across self-reported ratings of overall health ratings while Cohen’s *d* and AUC-ROC discriminations were used for opioid medication use and inability to work due to sickness or disability in the validation data. **c**, Longitudinal prognosis of secondary outcomes at about 9 years follow-up predicted from the risk score at baseline. Cohen’s *d* and AUC-ROC were measured in worsening at follow-up (left in red) and improvement (right in blue). *P* obtained using a two-sided unequal variance *t*-test (Welch’s approximation). Sample sizes are included in parenthesis. ATC, Anatomical Therapeutic Classification; N02A, Opioids ATC Classification.

**Extended Data Fig. 5:**
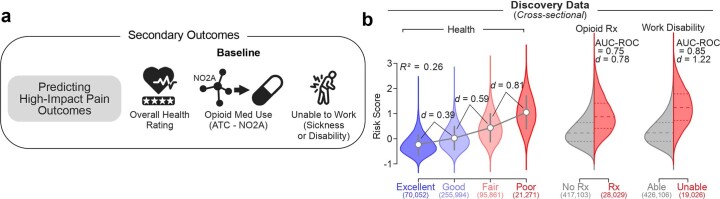
Examination of outcomes associated with high-impact pain in the discovery set. **a**. Schematic of a selection of three selected secondary outcomes. ATC, Anatomical Therapeutic Classification; N02A, opioids ATC Classification **b**. Cross-sectional performance of the risk score on the secondary outcomes in the discovery data. Cohen’s *d* effect sizes and explained variance (R^2^, on the left) were used across self-reported ratings of overall health ratings while Cohen’s *d* and AUC-ROC discriminations were used for opioid medication use and inability to work due to sickness or disability. Cohen’s *d* effect sizes and AUC-ROC discriminations were used. Sample sizes are included in parentheses.

The risk score for pain was also generalized to secondary pain outcomes (Fig. 4a), including overall health rating (*R*^2^ = 0.20, *P* < 1.0 × 10^−300^; Cohen’s *d* between consecutive categories of 0.38–0.78), use of opioids (AUC 0.73; Cohen’s *d* = 0.72) and disability due to sickness (AUC 0.88; Cohen’s *d* = 1.35; Fig. 4b). The longitudinal analyses demonstrated that the risk score for pain predicted the initiation of opioid medication (AUC 0.67; Cohen’s *d* = 0.51) and the development of disability (AUC 0.78; Cohen’s *d* = 0.94; Fig. 4c), which echoes the associations previously observed between high-impact pain and overlapping pain conditions (Fig. 1). Concordant results were observed in the discovery set (Extended Data Fig. 5). Overall, our results show that the risk score for pain also predicted high-impact chronic pain.

### Pain risk score and biological markers

**Extended Data Fig. 6:**
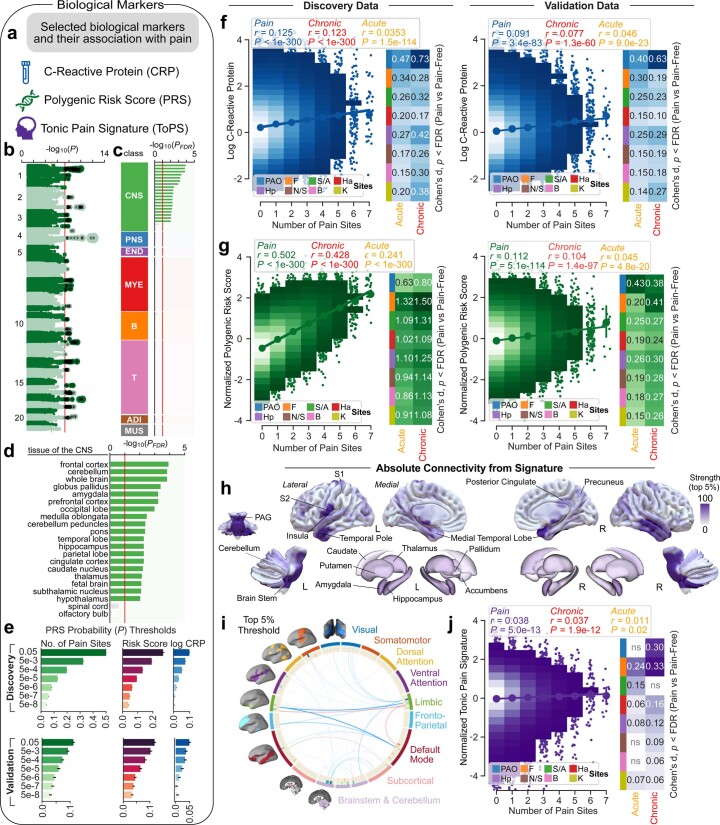
Examination of three prospective biological makers and their associations with pain. **a**. Schematic describing the selected biological markers: c- reactive inflammatory protein, a polygenic risk score for the number of pain sites, and a validated brain signature for sustained pain. **b-e**. Genome-wide association study of number of pain sites in the discovery data. **b**. The Manhattan plot shows association -log10 (P) for each single nucleotide polymorphism. **c**. The partitioned heritability in tissues of the Benita et al. dataset is shown for 78 tissues grouped into eight tissue classes: central nervous system (CNS), peripheral nervous system (PNS), endocrine (END), myeloid (MYE), B cells (B), T cells (T), adipose (ADI) and muscle (MUS). P-values were FDR-adjusted (10%) for enrichment with significant tissues colored. **d**. Details shown for the CNS tissue class**. e**. PRS repeated across an array of thresholds, with the least stringent threshold taken to maximize prediction. Associations between each threshold with the number of pain sites, risk score and CRP is shown and estimated using a two-tailed Pearson’s *r* correlation (standard error are estimated from 1,000 bootstrap samples). **f**. Two-tailed Pearson’s *r* correlation was also used to assess the association between CRP (log- transformed for parametric estimations) and the number of pain sites in both discovery (*P* < 1.0e-300) and validation (*P* < 3.4e-83) datasets. **g**. The association between the selected PRS and the number of pain sites in both discovery (*P* < 1.0e-300) and validation (*P* < 5.1e-114) datasets. Ha, headache; F, facial; N/S, neck or shoulder; S/A, stomach or abdominal; B, back; Hp, hip; K, knee. **h**. Visualization of the absolute connectivity from the Tonic Pain Signature (ToPS) computed from resting-state functional Magnetic Resonance Imaging (rsfMRI) and thresholded for the top 5% of weights. Represents the sum of normalized dynamic conditional correlation connectivity across each brain parcel. PAG, periaqueductal gray; S1, primary somatosensory cortex; S2, secondary somatosensory cortex; L, left; R, right. **i**. Circular graph representing the links of the computed ToPS across each major brain networks. **j**. The association between the ToPS (top 5% weights) and the number of pain sites in the validation (*P* < 5.0e-13) dataset. The Cohen’s *d* effect sizes for each marker are presented for each pain site compared to pain-free individuals. Comparisons were FDR-corrected (q < 0.05, ns > 0.05).

**Extended Data Fig. 7:**
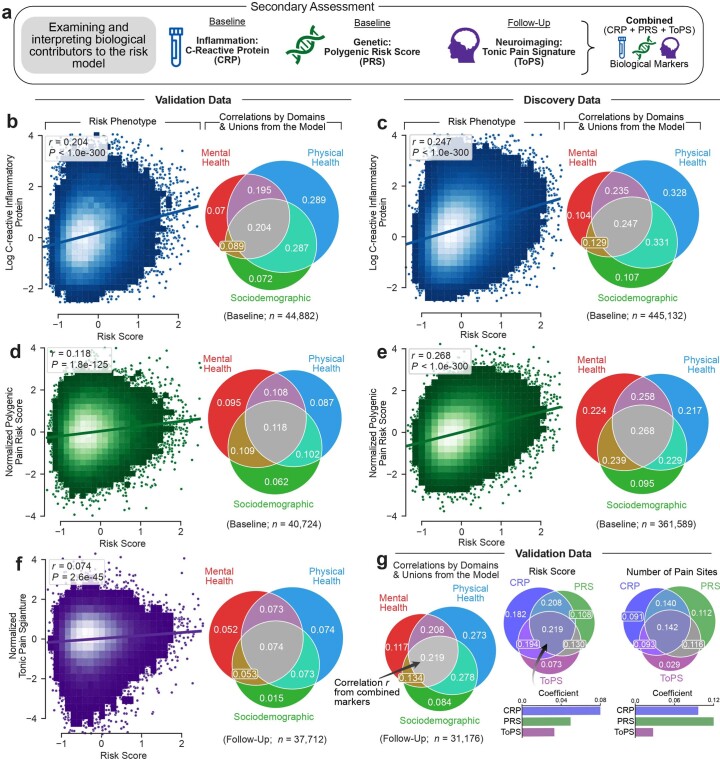
Inflammatory, genetic, and functional connectivity markers associated with the risk score for pain. **a**. Schematic describing the selected biological markers: c- reactive inflammatory protein, a polygenic risk score for the number of pain sites, and a validated brain signature for sustained pain. CRP, C-reactive protein; PRS, polygenic risk score; ToPS, Tonic Pain Signature. **b-c**. Two-tailed Pearson’s *r* correlation the association between CRP (log-transformed for parametric estimations) and our risk score in both **b**. validation set (*P* < 1.0e-300), and **c**. discovery set (*P* < 1.0e-300). **d, e**. The association between the selected PRS and our risk score in both **d**. validation set (*P* < 1.8e-125), and **e**. discovery set (*P* < 1.0e-300). **f**. The association between the ToPS and our risk score in the validation set (*P* < 2.6e-45). Venn diagram shows the correlation between the biological measures with respect to the three domains and their unions**. g**. Markers were combined as one variable and examined in the validation set. The respective contribution of biological markers to pain risk score and the number of pain sites are reported in the Venn diagrams.

We next tested whether certain biological markers were associated with the risk score for pain (Extended Data Fig. 6). The markers included C-reactive protein (CRP), a polygenic risk score (PRS) and a validated brain-based biomarker called the tonic pain signature (ToPS)^30^. These markers showed small but significant associations with the number of pain sites in the validation dataset (CRP, *r* = 0.09, *P* = 3.4 × 10^−83^; PRS, *r* = 0.11, *P* = 5.1 × 10^−114^; ToPS, *r* = 0.038, *P* = 5.0 × 10^−13^), with equivalent or even stronger correlations found with the risk score for pain (CRP, *r* = 0.20, *P* < 1.0 × 10^−300^; PRS, *r* = 0.12, *P* = 1.8 × 10^−125^; ToPS, *r* = 0.074, *P* = 2.6 × 10^−45^; Extended Data Fig. 7). This supports the idea that psychosocial factors are connected to biological factors in the experience of pain, as suggested by the biopsychosocial model for pain^5^. Additional analyses integrating domains for each biological marker or their combinations, are presented in Extended Data Fig. 7.

### Pain-specific candidate models for different body sites

**Fig. 5:**
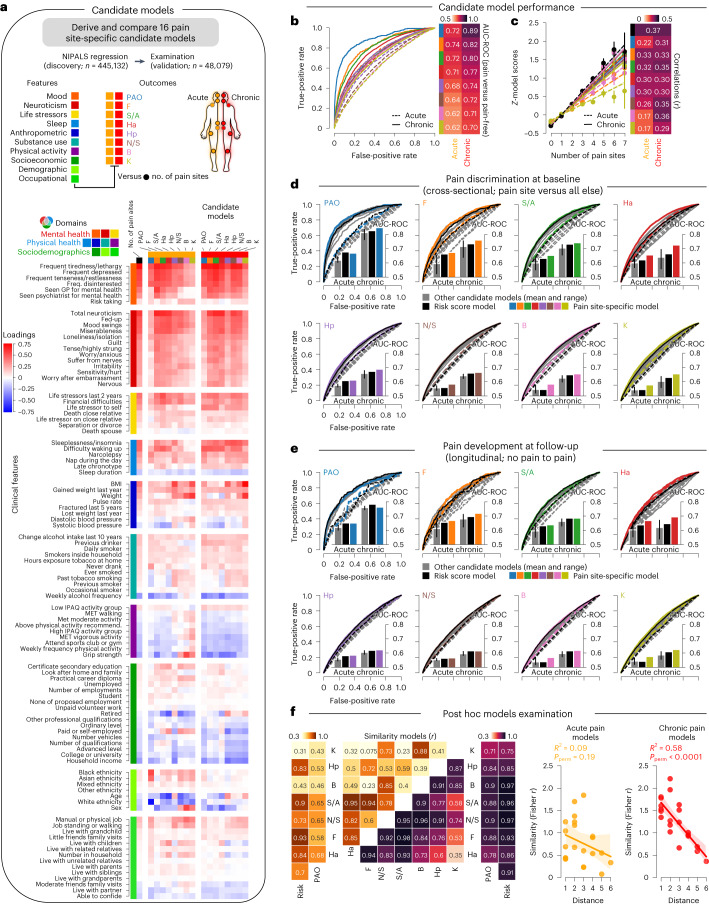
A common risk shared across chronic pain conditions. **a**, Schematic describing that a total of 16 site-specific candidate models (for example, acute knee versus all else) were derived cross-sectionally in the discovery set using NIPALS. Feature loadings (Pearson’s *r* correlation coefficient between features and the models’ scores) are shown in the testing set for each model. IPAQ, International Physical Activity Questionnaire; MET, metabolic equivalent task. **b**, Candidate models’ capacities to discriminate between the pain sites they were trained on from pain-free individuals are shown using AUC-ROC. **c**, The risk score derived from each candidate model correlated with number of coexisting pain sites for acute and chronic pain conditions separately (risk scores presented as mean ± s.e.m. estimated from 10,000 bootstrap samples, *n*_chronic_ = 17,948; *n*_acute_ = 13,117). **d**, Cross-sectional discrimination for each pain site in acute (dashed line) and chronic (full line) pain conditions against the rest of the testing cohort (pain-free and other pain sites) using the model specific to the site (in color), to the number of pain sites (black) and to other candidate models trained on different pain sites (gray). **e**, The same analyses were performed in the longitudinal data to predict the development of chronic pain in pain-free individuals about 9 years later. **f**, Post hoc analyses show that similarities between the feature loadings the different models (Fisher-normalized) can be explained (*R*^2^) by the distance between the sites in chronic (*P*_perm_ < 0.0001), but not acute pain conditions (*P*_perm_ = 0.19, using 10,000 two-sided permutation tests). The 95% CI estimated across 1,000 bootstrap samples is shown.

**Extended Data Fig. 8:**
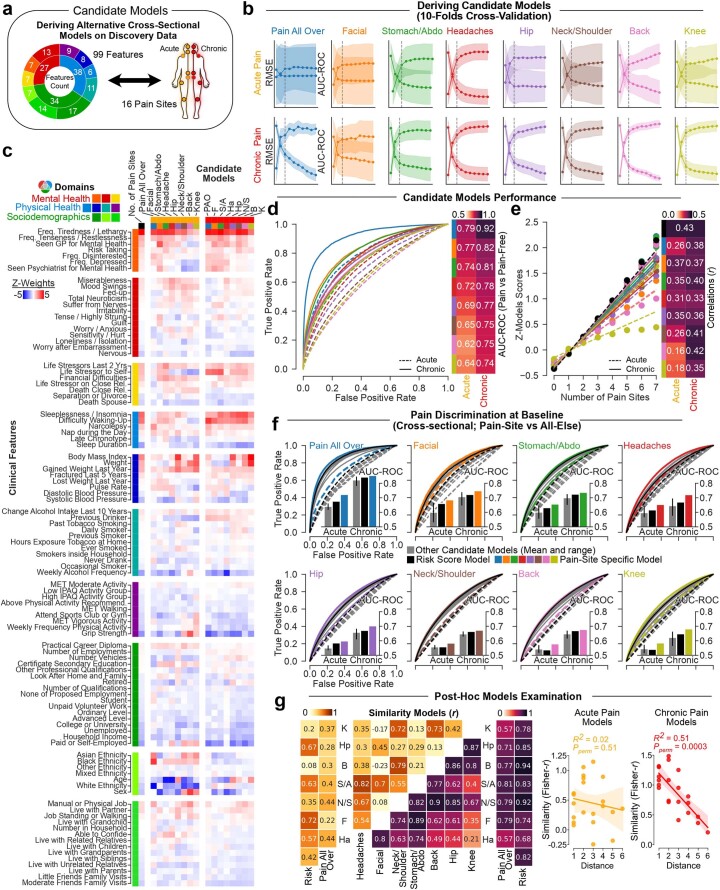
Deriving candidate models for chronic and acute pain conditions. **a**. Schematic describing the 99 features to derive a total of 16 site-specific candidate models cross-sectionally in the discovery set**. b**. tenFold cross-validation was used to estimate the root mean squared error (RMSE) and explained variance (R^2^). The same number of components were used to ensure comparability between derived models using NIPALS. **c**. Weights used for each model (normalized to allow comparison) grouped across categories and domains. Ha, headache; F, facial; N/S, neck or shoulder; S/A, stomach or abdominal; B, back; Hp, hip; K, knee. **d**. Candidate models’ capacities to discriminate between the pain sites they were trained on from pain-free individuals are shown using AUC-ROC. **e**. The risk score derived from each candidate model correlated with number of co-existing pain sites. **f**. Cross- sectional discrimination for each pain site in acute (dashed line) and chronic (full line) pain conditions against the rest of the training cohort (*that is*, pain-free and other pain sites) using the model specific to the site (in color), to the number of pain sites (black), and to other candidate models trained on a different pain site (gray). **g**. Post-hoc analyses show that similarities between the weights of the different models (Fisher-normalized) can be explained (*R*^*2*^) by the distance between the sites in chronic (*P*_perm_ = 0.0003) but not acute pain conditions (*P*_perm_ = 0.51, using 10,000 two-sided permutation tests).

We next investigated the specificity of the risk factors between different pain conditions by generating and testing alternative candidate models for each pain site separately. The 16 new candidate models were trained by applying the NIPALS algorithm on the 99 features to classify each body site separately (for example, participants reporting chronic knee pain versus everyone else). The matrix presented in Fig. 5a shows the loadings of the 99 features on the risk score derived for different pain conditions, including our initial model predicting the number of coexisting pain sites. A visual inspection of the matrix shows that the most predictive features for the spreading of pain were also the most homogenous between pain conditions. The models trained to classify acute pain conditions showed poor to good discrimination (AUC 0.62–0.74), whereas the models trained to classify chronic pain conditions showed good to excellent discrimination (AUC 0.70–0.89; Fig. 5b). The expression of each risk score (normalized for comparisons) correlated with the number of coexisting pain sites (Fig. 5c). The weights of the 99 features are presented for each candidate model in Extended Data Fig. 8.

To test the commonality or the specificity between the body sites, we compared the discriminative value of each site-specific model with that of the candidate models trained on a different pain site, in both cross-sectional (Fig. 5d) and longitudinal (predicting the development of chronic pain; Fig. 5e) data. In both cases, site-specific models showed only modest improvement over the risk score for pain (improvement in AUCs ranged from 0.006–0.065 in cross-sectional data and −0.021–0.047 in longitudinal data) and models trained for a different body site (improvement in AUCs ranged from 0.024–0.085 in cross-sectional data and 0.004–0.074 in longitudinal data). Similar results were observed in the discovery dataset (Extended Data Fig. 8). We conclude that different chronic pain conditions can be predicted from interchangeable models trained on a different pain site. These findings support the proposition that a common framework may be used to characterize different pain conditions that tend to co-occur and be predicted from the same features. The main difference in the performance of the model was instead that the risk score for pain discriminated between participants reporting pain at one body site and participants reporting pain at more than one body sites (chronic pain AUC 0.68–0.75; acute pain AUC 0.57–0.63; Supplementary Fig. 2).

A similarity matrix correlating the loadings between the candidate models showed that our initial model trained on the number of pain sites loads strongly on each of the eight candidate models trained for specific chronic pain conditions (*r* = 0.75–0.97; Fig. 5f). The similarities between the chronic pain candidate models depended on the distance between the body sites, reflecting the actual body map of the coexisting pain sites presented in Fig. 1b (*R*^2^ = 0.58, *P*_perm_ < 0.0001). This was, however, not the case for acute pain conditions, as the candidate models loadings onto the risk score varied between body sites (*r* = 0.31–0.93) and did not depend on the distance between pain sites on the body map (*R*^2^ = 0.09, *P*_perm_ = 0.19). Consistent results were obtained in the discovery dataset (Extended Data Fig. 8).

### Risk of pain spreading screening

**Fig. 6:**
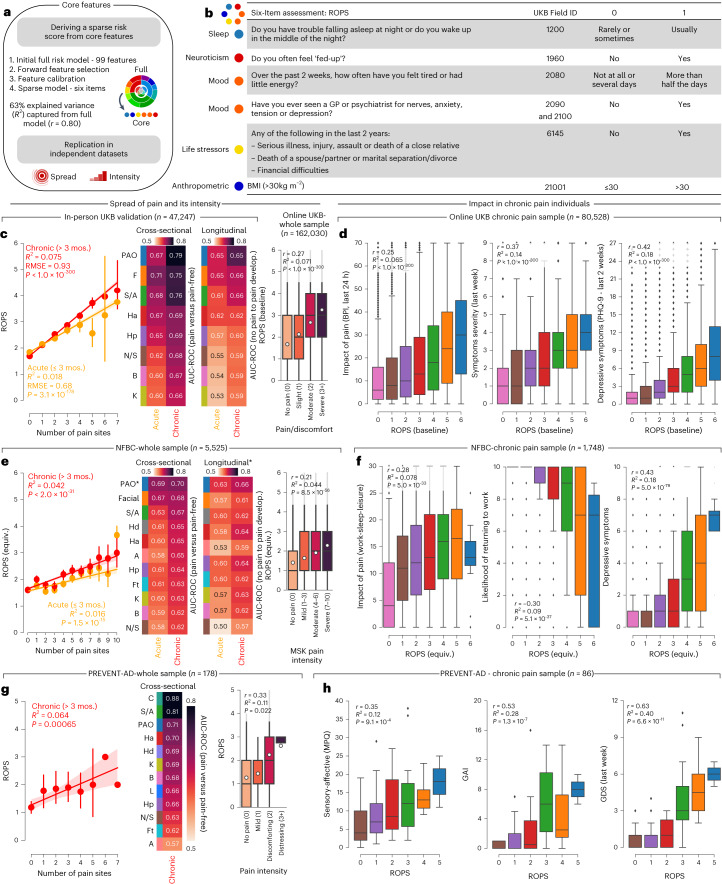
The risk of pain spreading screening. **a**, Schematic describing the steps implemented to develop the ROPS on 459,855 participants. **b**, Core selected features retained and binarized to form a six-item short score capturing 63% of the variance explained by the full risk score predicting the number of pain sites. **c**, Model performance on the testing set for the number of pain sites in both acute and chronic pain sites in the cross-sectional (*n*_chronic_ = 17,948, *n*_acute_ = 13,117) and longitudinal data and with pain intensity during the online assessment. **d**, In the online pain assessment, the ROPS was associated with the interference of pain, symptom severity during the last week and the depressive symptoms severity in last 2 weeks (*n*_ROPS:0_ = 9,794, *n*_ROPS:1_ = 18,460, *n*_ROPS:2_ = 20,102, *n*_ROPS:3_ = 16,489, *n*_ROPS:4_ = 10,423, *n*_ROPS:5_ = 4,349 and *n*_ROPS:6_ = 911). **e**–**h**, These results were replicated in independent cohorts including the NFBC cohort (using equivalent score items, longitudinal-only sample, *n* = 4,710) and the PREVENT-AD cohort (using identical score items). Hd, hand; A, arm; Ft, feet; C, chest; L, leg; GAI, geriatric anxiety scale; GDS, geriatric depression scale. In the NFBC, the ROPS predicted the number of pain sites and classified different pain conditions in both cross-sectional (*n* = 5,525) (*n*_chronic_ = 1,489; *n*_acute_ = 2,374) and longitudinal data (*n* = 4,710) with similar accuracy as in the UKB (**e**). The ROPS determined impact, working disability and depressive mood in the NFBC cohort (*n*_ROPS:0_ = 334, *n*_ROPS:1_ = 413, *n*_ROPS:2_ = 408, *n*_ROPS:3_ = 344, *n*_ROPS:4_ = 184, *n*_ROPS:5_ = 62 and *n*_ROPS:6_ = 12) (**f**). In the PREVENT-AD cohort, the ROPS predicted the number of pain sites and classified different pain conditions in cross-sectional data (**g**). The ROPS determined sensory and affective pain measured with the MPQ, anxiety and depressive mood (*n*_ROPS:0_ = 13, *n*_ROPS:1_ = 29, *n*_ROPS:2_ = 12, *n*_ROPS:3_ = 22, *n*_ROPS:4_ = 8 and *n*_ROPS:5_ = 2) (**h**). Box plots show the medians and are bound by the first and third quartiles. Data points outside 1.5 × interquartile range are shown as diamonds. PAO in both replication cohorts was defined as pain in five or more sites. The 95% CI was estimated across 1,000 bootstrap samples (**c**,**e**,**g**). In boxplots, the center line (median), white dot (mean), box (inner quartiles), whiskers (bottom and top bounds) and diamonds (outliers outside 1.5 × interquartile range) are shown. MSK, musculoskeletal. *Longitudinal data had a different *n* than the whole sample.

**Extended Data Fig. 9:**
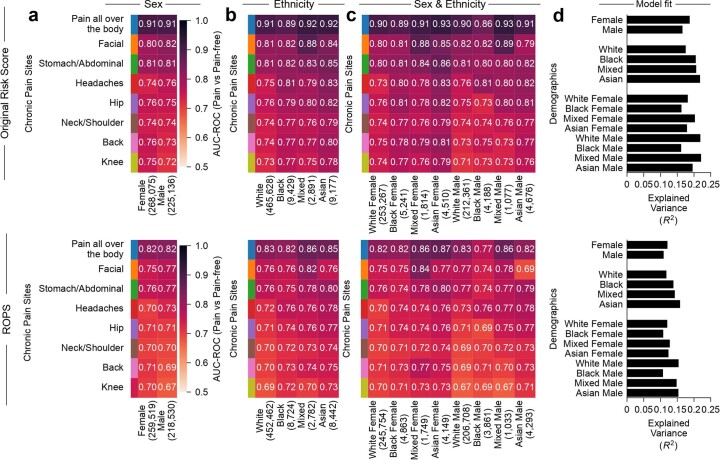
Minimal demographic bias in the original risk score and the Risk Of Pain Spreading. Cross-sectional AUC-ROC discrimination of chronic pain sites compared to pain-free individuals within **a**. females and males separately, **b**. white, black, mixed, and Asian ethnicities and **c**. each ethnicity and sex interaction. **d**. Model fit using explained variance (*R*^*2*^) for both risk scores across each demography is shown. Sample sizes are reported in parentheses from the entire UK Biobank cohort.

Last, we aimed to simplify our model by reducing the number of features (Fig. 6a). We re-trained the model by incrementally adding the items explaining the most variance. This simplified model, named the risk of pain spreading (ROPS), is a new risk score for pain calculated by simply summing the binarized answers to six items measuring sleep, neuroticism, mood, trauma and anthropometric measurements (Fig. 6b). The ROPS was associated with the number of pain sites (*R*^2^ = 0.075, *P* < 1.0 × 10^−300^) as well as pain intensity (*R*^2^ = 0.071, *P* = 8.5 × 10^−56^) and achieved good performance in cross-sectional data (chronic pain AUC 0.66–0.79; acute pain AUC 0.60–0.71) and average-to-good performance in longitudinal data (chronic pain AUC 0.59–0.73; acute pain AUC 0.53–0.65, Fig. 6c). This represented the best trade-off between the smallest number of features (obtained using the most predictive features from the original 99 features) and the highest AUC receiver operating characteristic (ROC), especially in the longitudinal data (chronic pain all over the body AUC 0.73). The ROPS predicted high-impact pain, as measured by pain interference (*R*^2^ = 0.065; *P* < 1.0 × 10^−300^), pain severity (*R*^2^ = 0.14; *P* < 1.0 × 10^−300^) and depressive mood (*R*^2^ = 0.18; *P* < 1.03 × 10^−300^; Fig. 6d). Furthermore, the original risk score and the ROPS both performed well across sexes (self-identified male or female, ROPS AUC 0.67–0.82 with sex differences in AUCs ≤ 0.03) and ethnicities (self-identified as White, Black, Asian or mixed, ROPS AUC 0.69–0.86 with ethnic differences in AUCs ≤ 0.06; Extended Data Fig. 9). None of these six features was directly related to pain or attitudes toward pain, suggesting that more objective predictions can be obtained by avoiding the use of pain questionnaires to predict pain outcomes.

**Extended Data Fig. 10:**
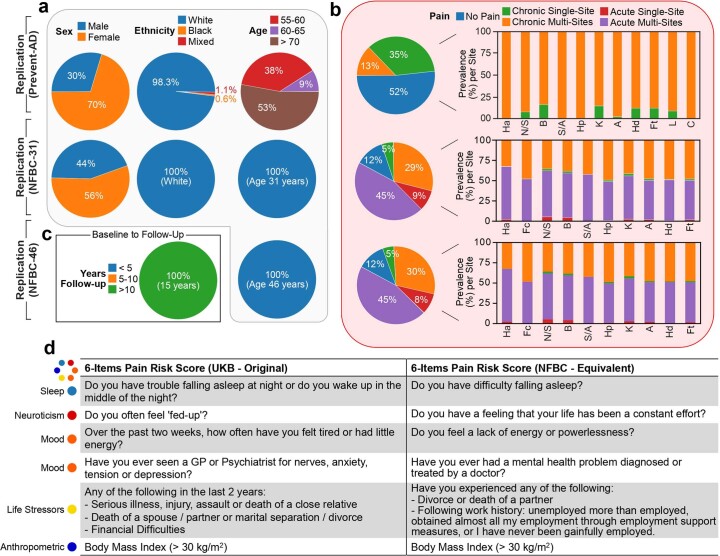
Validation of the ROPS in two independent cohorts. Pie charts displaying **a**. demographics**, b**. acute (≤3 months) and c. chronic (>3 months) pain phenotypes for the replication data, shown in the Prevent-AD in the top row, and in the NFBC at 31 and 46 years old shown in the second and third rows respectively. NFBC, Northern Finland Birth Cohort; Ha, headache; F, facial; N/S, neck/shoulder; B, back; S/A, stomach/abdominal; Hp, hip; K, knee; A, arm/elbow; Hd, hand; Ft, feet; C, chest; L, leg. **c**. Years between baseline (31 years old) and follow-up visit (46 years old) in the Northern Finland Birth Cohort data (15-year follow-up). **d**. Equivalence of the six-item pain risk score (ROPS) in the NFBC to the original from UK Biobank, also used in Prevent-AD.

We then tested the ROPS using the Northern Finland Birth Cohort (NFBC), which includes 5,525 participants born in 1966, 4,710 of whom were followed longitudinally. This cohort was selected because equivalent items were found for each one of our six items, with pain phenotype and demographics similar to the UK Biobank (Extended Data Fig. 10). In the NFBC, the ROPS predicted pain cross-sectionally (chronic pain AUC 0.62–0.70; acute pain AUC 0.58–0.69) and longitudinally (chronic pain AUC 0.57–0.66; acute pain AUC 0.50–0.63) with similar accuracy to the score initially developed in UK Biobank (Fig. 6e). Moreover, the ROPS also partially determined the impact of pain (*R*^2^ = 0.044, *P* = 5.0 × 10^−33^), work disability (*R*^2^ = 0.078, *P* = 5.1 × 10^−37^) and depressive mood (*R*^2^ = 0.18, *P* = 5.0 × 10^−79^) in participants reporting chronic pain (Fig. 6f).

We also directly administered the ROPS to a smaller group of participants enrolled in the Pre-symptomatic Evaluation of Novel or Experimental Treatments for Alzheimer’s Disease (PREVENT-AD) cohort. These participants are older adults with normal cognitive function enrolled in a longitudinal study aimed to identify risk factors for Alzheimer’s disease. Participants only responded to the five questions displayed in Fig. 6b (BMI was calculated from previous health records), preventing any flexibility in the selection of feature equivalence entered in the predictive model. The results were almost identical to those observed in the UK Biobank and the NFBC for cross-sectional classifications (AUC 0.57–0.88; Fig. 6g), pain intensity (*R*^2^ = 0.11, *P* = 0.022), McGill Pain Questionnaire (MPQ) sensory-affective scales (*R*^2^ = 0.12, *P* = 9.1 × 10^−4^), anxiety (*R*^2^ = 0.28, *P* = 1.3 × 10^−7^) and depressive mood (*R*^2^ = 0.40, *P* = 6.6 × 10^−11^; Fig. 6h).

## Discussion

In this study, we trained a model to predict the number of pain sites and derive individual pain risk scores. These scores classified each chronic pain condition separately in cross-sectional data (seven different body sites and 25 pain-related medical conditions), forecasted individual differences in pain spreading or recovery after 9 years and generalized to secondary outcomes such as general health, disability and opioid use. To simplify the model’s practicality, we developed a user-friendly screening tool named the ROPS, restricted to only six questions with binarized answers. The ROPS effectively predicted widespread chronic pain and its impact across three different cohorts. Predicting chronic pain conditions has numerous potential applications, such as patient selection for research protocols, participant matching in randomized controlled trials and guiding treatment options for patients in urgent need.

Using a multivariate approach, we identified that major risk factors for co-occurring pain on multiple body sites were mood (tiredness and consulting GP for depression/anxiety), sleep (insomnia), neuroticism (feeling fed-up) and anthropometric measures (BMI). Mental health-related factors were consistent across different pain sites and were the strongest predictors, whereas sociodemographic factors were heterogeneous across pain sites and were least predictive of the number of chronic pain sites. The comparison between candidate models trained on different body sites showed little superiority to body site-specific models, indicating that different chronic pain conditions can be predicted cross-sectionally and longitudinally from common risk factors. Our findings suggest that the biopsychosocial model not only shapes pain experience and maintenance, but also predisposes the development of new pain sites, a phenomenon we refer to as the ‘spreading’ of pain sites.

An increasing number of conditions resembling widespread pain disorders have been referred to as chronic overlapping pain conditions (COPCs). Our results showed that co-occurring pain sites beyond the traditional focus areas (such as headaches in migraine, stomach/abdominal pain in irritable bowel syndrome (IBS) or hand pain in carpel tunnel syndrome) are common in conditions other than the traditional COPCs. Furthermore, we found that the pain site co-occurrence was not random, with a strong dependence between proximal pain sites, shown from either acute or chronic pain sites and from correlations between pain intensity ratings. Thus, biopsychosocial risk scores developed for headache will also moderately predict knee pain and vice versa, indicating insensitivity to specific pain sites, although the more distal the pain sites, the more dissimilar the models were. All candidate models were effective in predicting widespread pain and diagnoses with a high prevalence of multi-site pain, such as fibromyalgia, regardless of whether they were trained on the number of pain sites or specific body sites. This suggests that an elevated risk could be a pathway for the progression of widespread pain disorders and helps in understanding how pain spreads across multiple sites.

This study highlights the significance of pain spreading as a core concept in COPCs. Our results show that individuals with a higher pain risk score are prone to developing pain in multiple sites and the extent of pain spreading across the body is more important than the location of pain. Furthermore, our results indicate that pain spreading encompasses the concept of high-impact pain characterized by limitations in work, social and self-care activities leading to disability, opioid use and increased healthcare needs^31,32^. This denotes that the concepts of chronic pain spreading and high-impact pain seem intimately linked and predictable from higher-order biopsychosocial characteristics presented from our data-driven framework. Therefore, we assert that research and prevention strategies should not solely focus on addressing the transition from acute to chronic pain, as solely focusing on the temporal evolution of pain is an incomplete narrative. Instead, our results suggest that the spatial trajectory of pain, whether it remains localized to a specific body site or spreads to proximal and then distal sites, is a central factor in chronic pain syndrome. Thus, prioritizing the study of spatial evolution of pain once it becomes chronic is crucial due to the prevalence of COPCs, the dynamic changes they undergo over time, the predictability of their spreading patterns, their biological underpinnings and their pivotal role in determining the severity of high-impact pain.

The biopsychosocial model has been influential in the field of chronic pain, as any model focusing solely on any one of these domains would inevitably be inadequate^5,33^. In this study, we also investigated the association between our pain risk score with biological markers, including CRP (inflammatory marker), PRS (genetic risk score) and ToPS (a brain-based signature for tonic pain). Our results revealed consistent but small associations between these biomarkers and the number of pain sites; however, these biomarkers were equivalently or even more strongly correlated with pain risk scores than with the number of pain sites alone. These findings raise questions about the pathophysiology of chronic pain and suggest that incorporating psychosocial factors may be more effective in understanding the biological determinants underlying chronic pain conditions.

We finally aimed to make our risk score for pain more clinically relevant and useful by simplifying it to a set of six questions with binary answers (yes/no), suitable for over-the-phone administration or screening visit assessments. The ROPS allows for a quick assessment of the risk of developing or transitioning to more severe forms of chronic pain. The ROPS has multiple applications, including enhancing research power by targeting vulnerable patients, optimizing patient allocations in randomized controlled trials and improving pain management by identifying individuals at risk of severe chronic pain conditions that persist or worsen over time.

Our study has several limitations. First, the UK Biobank lacks diversity, being a predominantly white population of middle-aged and older individuals. This may restrict the applicability of our model, as studies have demonstrated that algorithms trained on mostly white participants can be mischaracterized in non-white participants^34^. We, however, found that the original score and the ROPS presented near identical discrimination between sex and ethnicities (Extended Data Fig. 9). Second, the UK Biobank may have a ‘healthy volunteer’ selection bias given the low participation of 5.45%^35^. Internal selection bias has been reported in the imaging visit (the validation cohort), where participants are sociodemographically similar (Extended Data Fig. 2) but generally healthier than participants from the baseline visit (the discovery cohort)^36^. We, however, generalized the risk score for pain in independent cohorts with different characteristics. Third, our study did not account for medical comorbidities or treatments when developing the risk score for pain, focusing instead on the association between higher risk scores and the diagnosis of medical conditions and/or medication use. Future research should explore the independent contribution of medical factors to chronic pain. Last, like any multivariate model, the weights of our model cannot be directly interpreted. We, however, estimated feature importance through loadings and interpreted the univariate associations between each feature and the risk score.

In conclusion, our model predicted chronic pain spreading across multiple body sites in nearly 50,000 out-of-sample individuals. We showed that high sensitivity and specificity could still be obtained for certain chronic pain conditions using only six questions. The ability to predict chronic pain, particularly COPCs and its severe forms, with minimal effort has the potential to benefit both research and clinical practice.

## Methods

### Overview of the UK Biobank population

The UKB project is a large-scale, prospective and ongoing study initially established to allow extensive investigation of genetic factors and lifestyle determinants of a diverse range of common diseases in middle-aged and older adults^37^. To recruit the intended sample size of approximately 500,000 participants, over 9 million invitations were sent to individuals registered in the UK National Health Service aged 40–69 years old and living within a reasonable distance from an assessment center. Baseline recruitment and data collection from 503,317 participants who consented to join the study took place between 2006 and 2010 in 22 assessment centers throughout Scotland, England and Wales. Subsets of baseline participants were invited later for follow-up visits and/or were asked to provide data on online questionnaires at certain time points. The following datasets from different time points are used to address different aims of our study.

#### Baseline UK Biobank (in-person assessment visit, 2006–2010, *n* = 502,494)

Data collection at recruitment included (1) a touchscreen questionnaire, where participants provided information on their sociodemographic, lifestyle, psychosocial factors (social support and mental health), as well as their health and medical history; (2) a verbal interview by a trained nurse, including data on early life factors, employment, medical conditions, medications and operations; (3) physical measurements; and (4) biological sampling. For the purposes of this study, our study sample was restricted to 502,494 participants that contained data available at the date of our data request.

#### Imaging UK Biobank (imaging follow-up visit, 2014–2020, *n* = 49,001)

A subsample of baseline participants was invited to attend a follow-up visit 3–13 years later (median 9 years). This visit included an MRI scan on the brain as well as the same questionnaires and assessments as the baseline^38^. After exclusion based on both visits, the sample for this follow-up visit was restricted to the 48,079 participants. More details regarding brain imaging is provided in Methods, Brain MRI measures.

#### Online UK Biobank (online pain questionnaire, 2019–2020, *n* = 167,255)

Additional assessments in a subset of participants recruited at baseline were conducted by the UKB using online questionnaires. Experience of pain questionnaires were administered about 8–13 years (median 10 years) after the baseline visit to allow better phenotyping of individuals with chronic pain. A subset of 332,587 participants were sent invitations and 167,255 filled out the online questionnaire. Subsections were used to validate the in-person pain phenotype using the same anatomical sites asked at the UKB baseline visit (ten sections, https://biobank.ndph.ox.ac.uk/showcase/ukb/docs/pain_questionnaire.pdf). About 94,074 of these individuals reported pain or discomfort lasting for more than 3 months (chronic pain).

### UK Biobank participants and data exclusion

From the initial 502,494 participants from the baseline assessment visit (baseline UKB), those with more than 20% of features missing among the 99 features selected for the pain risk score (as explained in Methods, Feature selection for the pain risk score) were excluded, as were participants with missing data at any of the acute or chronic pain sites (equivalent to a total of <2.5% of the participants). These exclusion criteria were implemented to ensure high confidence regarding the number of pain sites, the primary outcome of this study. To ensure the findings of the study were as generalizable as possible to the greater population, no other exclusion criteria were applied. This resulted in a total study population of 493,211 individuals. The total study sample was then divided into a discovery cohort of 445,132 participants who did not attend the imaging follow-up visit and a validation cohort of 48,079 participants who did attend the imaging visit (imaging UKB). As the validation cohort includes participants from the imaging follow-up visit and longitudinal data are available for these participants, this subsample was used for the purposes of longitudinal analyses in this study.

To minimize potential bias from incomplete questionnaires, a data-driven Bayesian ridge regression model was applied for imputation of missing data as a function of all other features in the model, using the median as a prior. A median-only feature imputation method was also tried and presented congruent results. Features were then standardized across the participants by centering the mean to zero and scaling the variance to one. The same process (exclusion followed by an imputation for missing data and standardization with the same mean and variance) was applied separately for the validation dataset.

Of the participants from the online UKB, only those reporting chronic pain (as explained in Methods, Pain phenotype in the UK Biobank, *n* = 94,074) were included in this study. Participants reporting PAO were not exposed to a subset of the questionnaire and were therefore excluded from the related analyses. Similarly, participants missing data at any of the pain sites examined were excluded (in-person 7 or full 12 pain sites available).

### Pain phenotype in the UK Biobank

#### Pain phenotype: baseline and imaging UK Biobank

##### One-month pain

Participants were asked whether they experienced pain that interfered with their usual activities at any major anatomical sites (head, face, neck or shoulder, back, stomach or abdominal, hip, knee or PAO) in the last month. Participants who answered PAO could not choose any other pain sites. This category consists of both chronic and acute pain.

##### Acute and chronic pain sites

Participants who reported having a given pain site in the last month were then asked whether this pain at the given site had persisted for more than 3 months. This question was used to distinguish between a chronic pain site, one present for more than 3 months according to the classification from the International Association for the Study of Pain^20^ and an acute pain site, one present for 3 months or less.

#### Pain phenotype: online pain questionnaire (online UK Biobank)

##### Chronic pain

Participants were asked whether they were troubled by pain or discomfort either all the time or intermittently for more than 3 months. This was followed by a question asking about where they had experienced this pain or discomfort in the last three months. The options for pain sites were the head, face, neck or shoulders, chest, stomach and abdomen, back, hip, legs, knees, feet, arms, hands and PAO.

#### Length of pain or discomfort

The online questionnaire inquired about the duration of the pain or discomfort (3–12 months, 1–5 years or more than 5 years) for participants reporting chronic pain.

#### Pain rating in the last 24 h

Participants were asked to rate their pain over the last 24 h for each reported chronic pain site on a 0–10 scale (0, no pain and 10, as bad as it could be).

#### Worst pain rating experienced in the last 24 h

Participants were asked to rate the pain that bothered them most at its worst in the last 24 h, from 0 (no pain) to 10 (pain as bad as you can imagine). Only participants reporting chronic pain at a specific body site (not PAO) were exposed to this question.

#### Pain interference in the last 24 h

Using the BPI^39^, the impact of pain functioning was assessed across seven items, including general activity, mood, walking ability, normal work, relations with other people, sleep and enjoyment of life, each rated out of 10 (0, does not interfere and 10, completely interferes). This was assessed for the most bothersome pain and only participants reporting chronic pain at a specific body site (not PAO) were exposed to this question.

#### Depressive symptoms in the last 2 weeks

Presence or absence and the severity of current depression as a common comorbidity with chronic pain was assessed using the PHQ-9 (ref. ^40^), where 0–4 indicates no/minimal depression severity and 5–9 indicates mild depression severity).

#### Symptom severity over the last week

Participants were asked to indicate the level of severity they experienced over the past week across three symptoms of the fibromyalgia symptom severity scale, including fatigue, sleep quality and cognitive symptoms (0 indicates no problem and 3 indicates severe, pervasive, continuous, life-disturbing problems).

#### Pain or discomfort today

Participants were asked to describe their health ‘today’ (choosing one of the following options: no pain/discomfort, slight pain/discomfort, moderate pain/discomfort, severe pain/discomfort or extreme pain/discomfort).

### Feature selection for the risk score for pain

A total of 99 features collected at the baseline in-person visit (baseline UKB) were selected a priori based on their relevance to chronic pain. The selection was based on the Prognosis Research Strategy (PROGRESS) group that recently provided a framework for the development of a prognostic model to determine risk profile^41^. Variables were organized through an iterative approach along a hierarchical framework from 99 variables into ten categories forming three distinct domains (mental health, physical health and sociodemographics). The three domains are as follows:

#### Mental health

The mental health domain includes three categories (1) neuroticism (all individual items and their total sum-score) based on 12 neurotic behaviors closely linked to negative effect; (2) traumas (illness, injury, bereavement or stress in the last 2 years) including six events; and (3) mood (reported frequency of certain moods in the past 2 weeks and visits to a GP or psychiatrist for nerves, anxiety, tension or depression).

#### Physical health

The physical health domain includes four categories (1) physical activity based on MET scores computed using the IPAQ^42^; (2) sleep, such as questions regarding duration, napping, snoring and sleeplessness; (3) substance use, such as smoking and alcohol use; and (4) anthropometric measures such as BMI, fractures that occurred over the last 5 years and blood pressure.

#### Sociodemographics

The sociodemographic domain includes three categories (1) socioeconomic status, such as education completed, income and employment; (2) occupational measures, such as individuals present within household, social entourage and manual or physical job; and (3) demographics such as age, sex and ethnicity.

A full list of all pain risk score measures and their corresponding UKB data fields are provided in Supplementary Table 1.

### Diagnoses of pain-related medical conditions

#### Medical conditions collected at each visit: baseline and imaging UK Biobank

Participants underwent a verbal interview about past and current medical conditions, in which a trained nurse confirmed or amended the type of medical condition that the participant reported through the touchscreen questionnaire. If the participants were uncertain of the type of illness they had, they would describe it to the nurse who would attempt to place it within the coding tree or enter it as a free-text description to be subsequently matched to a specific entry by a doctor. Only noncancer illnesses were investigated.

#### Medical conditions collected online: online UK Biobank

The online pain questionnaire includes self-reported diagnoses of 14 common pain medical conditions. These included osteoarthritis affecting one or more joints, rheumatoid arthritis affecting one or more joints, cancer pain, carpal tunnel syndrome, complex regional pain syndrome, chronic post-surgical pain, diabetes, nerve damage/neuropathy, fibromyalgia syndrome, chronic fatigue syndrome or myalgic encephalomyelitis, gout, migraine, pelvic pain and post-herpetic neuralgia.

### High-impact chronic pain measurements

#### Overall health rating

The self-reported health rating was assessed through the touchscreen questionnaire at the baseline visit and the imaging follow-up visit (baseline and imaging UKB).

#### Use of opioid medication

Medication was obtained from self-reported regular use (most days of the week for the last 4 weeks) of prescription medications at the baseline visit and the imaging follow-up visit (baseline and imaging UKB) and was coded according to the ATC classification system of the World Health Organization obtained from the Wu et al. (2019) stratification^43^. Opioid medication use was extracted at each visit from the ATC-WHO coded data (ATC codes N02A and R05DA04).

#### Unable to work due to sickness disability

This measure was included as a section of the participants’ current employment status at the baseline visit and the imaging follow-up visit (baseline and imaging UKB).

### Overview of the replication study cohorts

#### NFBC replication

The NFBC1966 was originally composed of 12,068 newborns in 1966 representing 96.3% of births in the target region that year at the University of Oulu^44^. The data utilized for this study were obtained at the 31-year and 46-year follow-up visits conducted from 1997 to 1998 and 2012 to 2014, respectively^45^. Cross-sectional analysis was conducted at the 46-year follow-up with a final population of 5,525 and only participants with complete data in the required pain questionnaires were included. A longitudinal analysis of participants present at both the 31-year and 46-year visit was also conducted with a total population of 4,710.

#### PREVENT-AD replication

The PREVENT-AD dataset is an observational cohort composed of healthy individuals at risk of developing Alzheimer’s disease due to a first-degree family of Alzheimer’s disease. This sample originally consisted of 349 adults aged older than 60 years at baseline visit (between 2011 and 2017) who met the eligibility criteria of investigation explained elsewhere^46^. Cross-sectional analysis was conducted on data available from a total of 178 individuals who answered the most recent questionnaire (only participants with complete data in the required pain questionnaires were included).

### Pain phenotype in the replication cohorts

#### Pain phenotype: NFBC

At the 46-year follow-up participants were asked whether they have had ‘pain or aches in the last 12 months’ (question (Q) 46) of the complimentary questionnaire), at which body sites they experienced this pain and for how long. Pain lasting longer than 3 months was considered chronic. Pain lasting less than 3 months was considered acute. Facial and stomach/abdominal pain were collected in a separate questionnaire. Participants reporting both chronic pain on the previously mentioned questionnaire and pain at the stomach/abdomen (reported in Q96 of the background, lifestyle and health survey) were defined as having chronic stomach/abdominal pain. Similarly, participants with chronic or acute facial pain were defined as participants reporting chronic or acute pain in Q46 and jaw or face pain once a week or more (Q101 of the background, lifestyle and health survey). Healthy controls were defined as those who reported no pain over the last year. Self-reported pain intensity, impact of pain, likelihood of returning to work and depressive symptoms were recorded on a 0–10 scale. Participants also reported whether they had pain-related illnesses, such as fibromyalgia, diagnosed by a doctor. PAO was defined as reporting five or more pain sites.

At an earlier visit (31-year follow-up) participants reported frequency of pain or aches over the past year at a variety of body sites as well as frequency of headaches in the past week. Although these items were unable to differentiate acute from chronic pain sufferers, we utilized this item to select participants who never experienced pain at a given site during the 31-year visit and tracked who among these participants developed pain at the next follow-up visit 15 years later.

#### Pain phenotype: PREVENT-AD cohort

The experience of physical pain was evaluated using the MPQ^47^. Participants were asked whether they had suffered from chronic pain (any pain lasting more than 3 months) in the past year. If they answered ‘yes’ they were considered to have chronic pain. This was followed by a question asking about the area where their pain occurs on a complete body template divided into 50 anatomical areas, which for the purposes of this study were combined and categorized into 11 different pain sites (arm or elbow, hand, leg, foot, chest, buttock, knee, back, abdominal, neck or shoulder, and head). This phenotype was used to define PAO as five or more sites and/or report of fibromyalgia. Other measures were used, including present pain intensity scale, sensory and affective ratings of the pain experience from the MPQ, GAI, GDS, as well as demographic-specific questionnaires.

### Data analyses in the UK Biobank

#### Number of coexisting pain sites characterizing different chronic pain conditions

In Fig. 1c, the co-occurrence of acute and chronic pain sites was measured using ORs from the exponential function of a logistic regression coefficient estimated for each combination of sites (excluding PAO). As conducted by Khoury et al. (2022)^23^, each reported pain site was assigned a number 1–7 for the baseline UKB and 1–10 for the online UKB data, starting from the highest point toward the lowest point. Distances between sites were measured as the number of sites setting apart each combination of the corresponding numbers. Explained variance (*R*^2^) between the distance and logarithmic value of the ORs between sites was assessed. To ensure the significance of the association between co-occurrence and distance, our results were compared to a null model generated from 10,000 two-sided permutation tests, using two-sided tests as indicated in the figure legends.

The prevalence of pain sites among self-reported medical conditions was assessed for each pain site (Fig. 1d). The associations were independently assessed between the total number of pain sites experienced as a continuum (excluding PAO) and pain duration, worst pain rating, pain interference, depressive symptoms and symptom severity using two-sided Pearson’s *r* correlation test and explained variance (*R*^2^) are shown in Fig. 1e–i.

#### Developing the predictive model predicting number of chronic pain sites

A NIPALS^29^ regression algorithm (implemented using scikit-learn.org/) was used to derive an epidemiological model that explained the number of pain sites reported at the baseline visit.

Briefly, NIPALS identifies latent patterns that maximize the covariance between two matrices. Here, the NIPALS method was trained within the discovery dataset to identify latent scores (**Ε** and **Ζ**) and loadings (**Ρ** and **Η**) that maximize the covariance between a *(445504, 99)* matrix of standardized psychosocial features (*Χ*_*i*_) and a *(445504, 1)* vector of self-reported number of pain sites (**Υ**):

1. Compute singular vectors **μ**, **ν** (weights) of covariance matrix *C* = *Χ*^Τ^**Υ**
2. Obtain the latent scores **Ε** and **Ζ** by projecting *Χ* and **Υ** onto singular vectors **μ** and **ν**
3. Compute loadings **Ρ** and **Η** by iteratively regressing *Χ* onto **Ε** (power iteration)
4. Deflate *X* and **Y** using *Χ* + 1 = *Χ* - **ΕΡ**^Τ^ and **Υ** + 1 = Υ - **ΖΗ**^Τ^, respectively
5. Fit training (discovery) data *Χ* using the projection matrix **Ρ** to obtain latent space x̄ so that x̄ ***=*** *Χ***Ρ**
6. Use the latent space to predict left-out data *(47708,1*) **Y**_**v**_ using the coefficient matrix *β*∈*R*^*d*^^⨯*t*^${\boldsymbol{\beta }}$ such that **Y**_**v**_ = **X**_**v**_*ß*, where *X*_**v**_ denotes the *(47708, 99)* matrix of psychosocial features in the validation set

Further information on the implementation can be found at https://scikit-learn.org/stable/modules/cross_decomposition.html#cross-decomposition.

This model excluded individuals reporting PAO to avoid making assumptions about the equivalence of PAO and some number of pain sites experienced. This specific algorithm was chosen to reduce the 99 features into a few sets of distinct homogenous components associated with self-reported number of pain sites. A common rule of thumb in multiple regression suggests that the minimum ratio of sample size per variable is 10:1, with greater ratios equivalent to greater stability. Here, we observed a 4,500:1 ratio of sample size per variable giving us confidence in our stability. Tenfold cross-validation was used to assess the number of components to use in the model. A total of three components were selected based on the largest increase in the variance explained and the largest decrease in RMSE according to the elbow criterion. The model was then applied in the validation dataset.

In Fig. 2b,c the model’s explained variance (*R*^2^) in the number of pain sites was assessed by organizing the 99 features into ten categories and three domains (physical health, mental health and sociodemographics). The category contributing the least (occupational) was still significant compared to a null model generated from 10,000 two-sided permutations. The model fit for predicting the number of pain sites in the testing set was assessed using explained variance (*R*^2^) and RMSE; Fig. 2d,). The risk scores of individuals with each pain site were compared to the score of pain-free participants to examine the impact of acute and chronic pain sites using Cohen’s *d* effect sizes (pooled s.d.; Fig. 2e) and the AUC-ROC for discrimination (Fig. 2f). The AUC-ROCs were used to estimate model accuracy because they are (1) threshold-unspecific and (2) resilient to class imbalance, which is inherent to less frequent pain conditions or clinical outcomes. The performance of the model was also tested across 25 different medical conditions commonly associated with chronic pain using the same metrics: Cohen’s *d* and AUC-ROC (Fig. 2g). To ensure the robustness of the results in less frequent medical conditions, 10,000 bootstrap samples were performed to estimate the CI in the observed effect sizes. Statistics calculated in the discovery data to evaluate the model performance and model interpretation are shown in Extended Data Fig. 3.

#### Network analysis

Networks were estimated from partial correlations between pain and the ten categories (Extended Data Fig. 5d). Partial correlations were used to measure conditional dependence between categories (defined as nodes) while controlling for all other potential edges. The number of acute pain sites and chronic pain sites were integrated into the network to assess the relative contributions of our model’s categories on both pain types. The networks were constructed and studied at three different densities. A threshold was first applied to obtain a sparse model and conserve connections equivalent to a small effect size (partial correlation >0.1). An intermediate model was then constructed using a more liberal threshold equivalent to a very small effect size (partial correlation >0.05). A full model was finally constructed by including all the edges surviving Bonferroni correction. Nodes were placed using a force-displacement layout (spring layout, using a Fruchterman–Reingold algorithm) using qgraph^48^. Starting in a circular layout and through various iterations, more-connected nodes are placed closer together, whereas less-connected or negatively linked nodes are placed further apart. Finally, node-weighted centrality, a measure of the mean number of edges passing through each node, was computed to estimate the centrality of both categories and pain outcomes. The procedure was conducted on both the discovery and validation datasets.

#### Spreading and recovery of chronic pain

The prognostic value of the pain risk score to predict the development, persistence and worsening of chronic pain was assessed using the left-out participants in the validation for whom the longitudinal data were available. After examining the stability of number of pain sites (0–4 or more, including PAO; Fig. 3a), the association between the risk of chronic pain at each anatomical site at follow-up and the risk of chronic pain at each site at baseline was calculated using ORs from the exponential function of the logistic regression coefficients. Explained variance (*R*^2^) between the distance from site at baseline and logarithmic value of the ORs between sites was calculated (Fig. 3b). To ensure the significance of the association, our results were compared to a null model generated from 10,000 two-sided permutation tests. The risk of chronic pain at each anatomical site at follow-up was then examined by calculating the OR associated with one unit increase in the risk score for each chronic pain site at baseline (Fig. 3c).

Spreading was measured using the change in number of chronic pain sites (from −4 or less to 4 or more). To examine the prognostic value of our risk score, we regressed out the number of chronic pain sites (and their squared values) reported at baseline, age at baseline and years to follow-up from the risk score calculated at baseline. Making the score orthogonal to the baseline pain allowed us to interpret interindividual deviations in this adjusted score as risk of recovery or spreading of pain at the follow-up visit (Fig. 3d). Effect sizes (Cohen’s *d*) were computed for the adjusted pain risk score between individuals without chronic pain and individuals with chronic pain based on changes in the number of chronic pain sites (for example, +1 sites versus pain free). The AUC-ROCs were also used to estimate whether these changes in the number of pain site can be predicted based on the adjusted risk score at baseline (Fig. 3e). The same approach was also used to predict the development or recovery of medical conditions longitudinally (Fig. 3f).

A temporal ordering of predicted risk across the ten categories was performed after adjusting for the number of pain sites (and squared values) at baseline. The effects sizes were calculated for the adjusted risk score within each category between participants reporting chronic pain and pain-free participants for different rates of spreading (+1 sites versus pain free, +2 sites versus pain free, +3 sites versus pain free and +4 sites versus pain free) and recovery (−1 sites versus pain free, −2 sites versus pain free, −3 sites versus pain free and −4 sites versus pain free). The categories were then ranked based on the magnitude of their effect sizes, as illustrated in Fig. 3g, after adjusting for the FDR. The ranking was calculated using the absolute sum of effect sizes across all rates of spreading or recovery, providing a temporal progression of risk across categories from early-to-late pain site development.

#### Sex-based analyses

Sex was collected by the UKB from central registry at recruitment and were updated by the participant if necessary. For both the NFBC and PREVENT-AD, sex was self-reported. No exclusion was made based on biological sex or gender. Sex ratio was reported for each cohort and included in the full partial least squares model applied in the UKB. Sex-stratified analyses performed for the full model and the ROPS are shown in Extended Data Fig. 9.

### Exploratory analyses

#### High-impact pain

The cross-sectional and longitudinal performance of the model for predicting secondary outcomes associated with high-impact chronic pain was assessed using self-reported ratings of overall health, opioid medication use and inability to work due to sickness or disability. In Fig. 4b, model fit in predicting secondary pain outcomes was assessed using Cohen’s *d* effect sizes and explained variance (*R*^2^) across self-reported ratings of overall health and using Cohen’s *d* and AUC-ROC discriminations for opioid medication use and inability to work due to sickness or disability. The longitudinal changes in opioid use and changes in ability to work were also calculated using Cohen’s *d* and AUC-ROC discriminations (Fig. 4c). Statistics calculated in the discovery data are shown in Extended Data Fig. 4b.

#### Candidate models

The same statistical procedure (NIPALS) performed on the same 99 clinical features was used to derive 16 candidate models classifying acute or chronic pain sites (versus all else; Fig. 5). Model specification was performed through tenfold cross-validation to maximize the variance explained (*R*^2^) while minimizing the RMSE. This allowed us to decide on the sparsity of components to include in the models using the elbow criterion from the largest drop in explained variance. The same parameters (three components, used as regularization) were used to predict the pain sites using NIPALS. Features for each model were visualized using two methods (1) by computing the Pearson’s *r* correlation equivalent to the loadings of each feature onto their projected score or (2) by comparing the *z*-normalized weights used to obtain the projected score. The former approach was preferred for interpretability (Fig. 5a).

The sensitivity of these candidate models was evaluated using AUC-ROC discrimination in comparison to pain-free individuals. The specificity of these models was assessed by comparing their AUC-ROCs with the one of our initial pain risk score (black) and the one from other candidate models trained on another pain site (gray). This was performed cross-sectionally (Fig. 5d) and longitudinally (Fig. 5e). For the longitudinal analyses, we assessed the capacity of the risk score to predict the development of pain in pain free individuals at baseline.

Figure 5f shows a post hoc analysis that was performed to examine the similarity of the 99 loadings (or normalized weights) across models using Pearson’s *r* correlation coefficients. This approach allowed us to compare the similarity between risk factors for acute and chronic pain separately. The correlation coefficients were then normalized using *z*-Fisher transformations and the explained variance (*R*^2^) was calculated using the similarity between models and the distance between pain sites. This procedure was also performed in the discovery data shown in Extended Data Fig. 8d–g.

#### The risk of pain spreading

##### Deriving the ROPS in the UK Biobank

A simplified model containing six features was derived from the full risk model containing 99 features in the training cohort of the UKB baseline dataset (*n* = 445,132). We trained a linear forward feature selection algorithm, implemented using scikit-learn, to select the core six features that presented the highest explained variance based on the full risk model. Forward feature selection initially finds the one feature that maximizes a cross-validated score in an outcome of interest (number of pain sites). Then, a second feature is added and the procedure is repeated for a prespecified combination of features in a feature pool (99 features from the original model) until there is no improvement in the model’s performance. Here, six features provided the best trade-off between sparsity and variance explained.

The identified features were then calibrated based on thresholds that maximized the discrimination performance (AUC-ROC) between individuals reporting PAO in the baseline UKB data and those not reporting PAO (*n* = 159,663). This procedure allowed us to generate binarized scores for each feature that facilitated the use of such a screening tool. For example, we evaluated thresholds ranging from 0–40 in increments of 5 and found that a BMI threshold of 30 led to the highest discrimination performance. Thus, individuals with a BMI >30 were coded as a 1 and those <30 were coded as 0. This procedure was repeated for all features on a categorical or continuous scale (sleeplessness, tiredness and traumas). This led to the formation of a six-item short questionnaire (ROPS) capturing 63% of the variance explained by the full risk model. In Fig. 6c, the cross-sectional and longitudinal performance of the ROPS was assessed in the discovery set for acute and chronic conditions for the number of pain sites using explained variance (*R*^2^) and for group differences between pain and pain-free groups using AUC-ROC matrices. In Fig. 6d, the longitudinal performance of ROPS was evaluated in the online UKB data in a subsample of 80,528 participants calculating the association between baseline risk scores and each of interference of pain ratings (BPI), depressive symptoms in the last 2 weeks and the severity of symptoms using Pearson’s *r* correlation and *R*^2^ metrics.

##### ROPS replication: Northern Finland Birth Cohort

An equivalent to the ROPS was constructed at both the 31-year and 46-year time points in the NFBC to determine both the cross-sectional and longitudinal validity of the score. The score was derived using six binarized items:

1. Do you have difficulty falling asleep, quite a lot or very much?
2. Do you have a feeling that your life has been a constant effort, quite a lot or very much?
3. Do you feel a lack of energy or powerlessness, quite a lot or very much?
4. Have you had a mental health problem diagnosed or treated by a doctor?
5. Have you experienced any of the following:
  - Divorce
  - Death of a partner
  - The following work history: unemployed more than employed, obtained almost all my employment through employment support measures or I have never been gainfully employed
6. Measured BMI greater than 30

In Fig. 6e, the performance of the ROPS was assessed for number of acute and chronic pain sites using *R*^2^ and RMSE and AUC-ROC scores showed the model’s ability to differentiate between pain free individuals and pain participants as well as differentiating between individuals with pain diagnoses and individuals without diagnoses cross-sectionally. A ROPS model derived at the 31-year visit was then utilized to predict participants reporting no pain at the 31-year visit who will develop pain at the equivalent site at the 46-year visit (stomach pain was excluded in longitudinal analysis due to the absence of a sufficiently similar item in the 31-year visit).

The association between the ROPS derived at the 46-year visit and the number of pain sites was determined for acute and chronic pain patients separately, with healthy controls counting as 0 pain sites in each correlation. Pain intensity was binned into four groups (no pain (0), mild pain (1–3), moderate pain (4–6) and severe pain (7+)) and the correlation between pain intensity and the sparse risk score across participants was calculated using Pearson’s *r* and *R*^2^. In Fig. 6f, impact of pain, likelihood of returning to work and depressive symptoms were correlated with the ROPS score in chronic pain participants.

##### ROPS replication: PREVENT-AD

For the purposes of this study, the same six items of the ROPS (as shown in Fig. 5b) were administered to the participants from the PREVENT-AD cohort. As conducted in the UKB data, in Fig. 6g, the cross-sectional performance of the sparse model was assessed for predicting the number of pain sites using explained variance (*R*^2^). The AUC-ROC was then used to differentiate participants reporting chronic pain from pain-free participants, at each pain site. In Fig. 6h, the ROPS was also evaluated on pain intensity scores, affective and sensory ratings of the MPQ, the total score on the GDS and the total score of the GAI using Pearson’s *r* correlation and *R*^2^ metrics.

### Biological measures and analyses

#### Immune-inflammatory profile

The baseline assessment visit data (baseline UKB) include a complete blood count (https://biobank.ctsu.ox.ac.uk/crystal/crystal/docs/haematology.pdf). The sample handling and storage has been described by Elitt and Peakman^49^. For the purposes of this study, inflammation was estimated using CRP obtained through saliva samples and measured by immunoturbidimetric assay using a high-sensitivity analysis on a Beckman Coulter Analyzer. Immune cell count included neutrophils, platelets, reticulocytes, basophils, lymphocytes, eosinophiles and monocytes, most of which have been shown to be independently linked to chronic pain, the sickness response and associated depressive profile^50^.

A logarithmic transformation was applied to the raw measures of CRP to account for the positive skewness (https://biobank.ndph.ox.ac.uk/showcase/field.cgi?id=30710). The association between CRP and the number of pain sites was evaluated using Pearson’s *r* correlation and Cohen’s *d* effect sizes comparing each pain site with pain-free individuals (Extended Data Fig. 6). Pearson’s *r* correlation between CRP and the risk score was assessed in both discovery and validation datasets (Extended Data Fig. 7). The associations between specific immune cell counts and CRP, pain risk score and the number of pain sites are reported in Supplementary Fig. 3.

#### Genetics

Blood samples collected at the baseline visit (baseline UKB) allowed different types of assays to be performed, including genetic analyses. A genome-wide association study of number of pain sites, including both acute and chronic was conducted. A thresholding procedure was conducted across seven statistical thresholds of significance (from *P* = 5 × 10^−2^ to 5 × 10^−8^) for each single nucleotide polymorphism. The association of each threshold with the risk score, CRP and pain phenotype was also examined in the discovery and validation datasets (Extended Data Fig. 6).

Partitioned heritability in tissues was used to investigate the genetic architecture of our polygenic risk score. The top 1,000 most-enriched genes per tissue were extracted from the gene expression database features in Benita et al. (2010) using the computer program ‘ldsc’^51–53^. A total of 78 tissues grouped into eight tissue classes (central nervous system, peripheral nervous system, endocrine, myeloid, B cells, T cells, adipose and muscle) were examined for enrichment. The methodology is described in greater detail in our previous publication^23^. Pearson’s *r* correlation between our normalized polygenic score and the risk score was assessed in both discovery and validation datasets (Extended Data Fig. 7).

#### Brain MRI measures

Resting-state functional MRI data available from the imaging follow-up visit (imaging UKB) were obtained from the UKB. The data were based on the minimally preprocessed pipeline designed and carried out by the FMRIB group, Oxford University. The minimally preprocessed resting-state fMRI data from the UKB were analyzed using the following pre-processing steps: motion correction with MCFLIRT^54^, grand-mean intensity normalization, high-pass temporal filter, fieldmap unwarping and gradient distortion correction. Noise terms were identified and removed using FSL ICA-FIX. Full information on the UKB pre-processing is published^55^. Additional pre-processing included warping the image in native space to the 3-mm MNI template (FSL), despiking using 3DDespike (AFNI from Nipype), 6-mm kernel smoothing (Nilearn) and resampling to 3 mm (for storage purposes). A modified Brainnetome atlas^56^ was used to parcel the brain into 279 distinct regions to apply the weights from the ToPS^30^, a capsaicin-induced tonic pain signature of pain derived from the brain that was associated with both experimental and clinical pain. The modified atlas includes additional midbrain, brainstem and cerebellar regions.

Dynamic connectivity was estimated to derive ToPS using dynamic conditional correlation, which is based on generalized autoregressive condition heteroscedastic and exponential weighted moving average models (implemented by https://cocoanlab.github.io/tops/). The preprocessing aimed to be as similar as possible to the original ToPS study without diverging from the minimally preprocessed data from the UKB. The weights of the signature were thresholded to the top 5% to avoid overfitting and to minimize relation with head motion before the examination of the full dataset (early subsample of *n* = 200). Multiple thresholds (1, 2.5, 5, 10, 25, 50 and 100%) were also tested to ensure generalizability. Absolute connectivity from the signature for visualization and interpretation purposes was computed using the normalized sum of absolute connectivity values for each brain region within the top 5% threshold, using a cutoff of 100 as the maximum (Supplementary Fig. 4). Surface rendering was conducted using the Surf Ice tool (https://www.nitrc.org/projects/surfice/).

Two frameworks were evaluated to control for the effects of confounding variables (1) adjusting confounding variance that does not overlap with pain and (2) adjusting total confounding variance. The first approach allows the brain signature to be compared to other polygenic and inflammatory markers that were left intact given the research focus on prediction, whereas the second ensures that our results do not overlap with confounds commonly reported as higher in patients with pain, such as motion. Results were very similar in both approaches, with the former presenting slightly smaller probability values.

The MRI-based covariates included head motion (linear, squared and cubed), imaging site, position in the scanner and coil position (*x*, *y* and *z*, respectively). Both covariates and brain features were normalized to a mean of zero and variance of one unit across participants. To examine confounding variance that did not overlap with pain, the number of pain sites was regressed out from confounds. A confound-removal procedure, conducted on the original confounds or pain-regressed confounds was applied by deriving a multivariate regression model to predict each normalized brain feature as a function of the normalized confounds. The procedure was carried out for each of the brain features, making them strongly or perfectly orthogonal to confounds. Pearson’s *r* correlation value between our normalized ToPS and the risk score as well as across each domain from the model was assessed in the validation dataset (Extended Data Fig. 7). The results were further displayed for each one of the nine brain networks separately (Supplementary Fig. 5).

### Statistical analysis

Data pre-processing and statistical analyses were performed using Python v.3.7 (including Numpy (v.1.22.0), Pandas (v.1.3.5), Sklearn (v.1.0.2), Nilearn (v.0.9.0) and Nltools (v.0.4.5)) and R software (Qgraph (v.1.9.2)). Permutation tests (with 10,000 iterations) were used to test whether the associations assessed by calculating Pearson’s *r* correlation were significantly higher than a null association. We used bootstrap resampling with 10,000 iterations to indicate the estimated error in the Cohen’s *d* effect sizes. Tenfold cross-validation was used to obtain unbiased model performance results. In all analyses, significance was based on *P* < 0.05 for single testing and FDR < 0.05 for multiple testing. Further details of the statistical methods are specified in each relevant section above.

### Ethical approval

The UKB was approved by the National Information Governance Board for Health and Social Care and the National Health Service North West Multicenter Research Ethics Committee (ref. no. 06/MRE08/65). All participants gave written, informed consent and the study was approved by the Research Ethics Committee (no. 11/NW/0382). Further information on the consent procedure can be found at https://biobank.ctsu.ox.ac.uk/crystal/field.cgi?id=200. Each follow-up study of the NFBC1966 was evaluated by the regional ethical committee of the Norther Ostrobothnia Hospital District (EETTMK 94/11, 17.09.2012). The use of NFBC data is based on cohort participants’ written informed consent at their latest follow-up study. Participants in the PREVENT-AD cohort provided written informed consent to participate at each follow-up visit, including questionnaires and multimodal imaging assessments. Protocols, consent forms and study procedures were approved by McGill Institutional Review Board and/or Douglas Mental Health University Institute Research Ethics Board.

### Reporting summary

Further information on research design is available in the Nature Portfolio Reporting Summary linked to this article.

## Online content

Any methods, additional references, Nature Portfolio reporting summaries, source data, extended data, supplementary information, acknowledgements, peer review information; details of author contributions and competing interests; and statements of data and code availability are available at 10.1038/s41591-023-02430-4.

## Supplementary information

Supplementary Information Supplementary Figs. 1–5 and Supplementary Table 1.

Reporting summary

Supplementary Table 1 Pain risk score measures. *Each data field represents the fundamental block of data available in the UKB repository, identifying the results of a single question or measurement. Colors indicate the domain assigned to each measure: sociodemographics, mental health and physical health.

## Data Availability

All data provided from the UKB are available to other investigators online upon permission granted by www.ukbiobank.ac.uk. Restrictions apply to the availability of these data, which were used under license for the current study (project ID 20802). The NFBC data are available upon request from the University of Oulu, Infrastructure for Population Studies (https://www.oulu.fi/en/university/faculties-and-units/faculty-medicine/northern-finland-birth-cohorts-and-arctic-biobank). Permission to use the data can be requested for research purposes via an electronic material request portal (Greip). PREVENT-AD data can be accessed openly at https://openpreventad.loris.ca, whereas most of the other information that is sensitive by nature is accessible by qualified researchers at https://registeredpreventad.loris.ca.

## Code availability

Detailed code and annotation will be available at GitHub (https://github.com/EVPlab). The medication classification performed by Wu et al.^43^ can be found in supplementary data from the original article (https://www.nature.com/articles/s41467-019-09572-5). Code to extract the ToPS by Lee et al.^30^ can be found online (https://cocoanlab.github.io/tops/).
