# Supplementary material for: A prognostic risk score for development and spread of chronic pain
Source: Nat Med. 2023 Jul 6;29(7):1821–31. doi: 10.1038/s41591-023-02430-4 (PMC10353938; doi:10.1038/s41591-023-02430-4)
Supplement: Supplementary file 1 — Supplementary Figs. 1–5 and Supplementary Table 1. [file 41591_2023_2430_MOESM1_ESM.pdf]

nature medicine

Article https://doi.org/10.1038/s41591-023-02430-4

A prognostic risk score for development and spread of chronic pain

In the format provided by the authors and unedited

SUPPLEMENTARY RESULTS

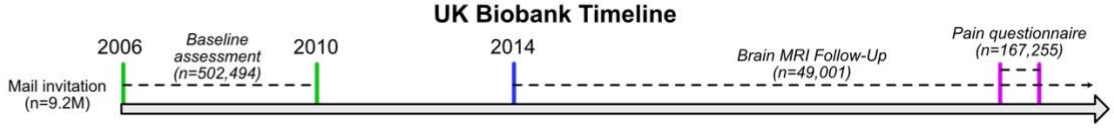

Supplementary Fig. 1 | Description of the UK Biobank timeline. Overview of the UK Biobank recruitment and timeline. Baseline assessment (in-person visit) was done between 2006-2010, N = 502,494) including a touchscreen questionnaire on sociodemographic, lifestyle and other health measures, a verbal interview by a trained nurse, physical measurements, and biological sampling. The brain MRI session, corresponding to the imaging follow-up visit (2014-2020, N = 49,001) with the same questionnaires as the baseline assessment is also shown. The pain questionnaire was administered online (2019-2020, N = 167,255). A subset of 332,587 participants were sent invitations, and 167,255 filled out the online questionnaire. More details can be found in the *Method – Overview of the UK Biobank Population*.

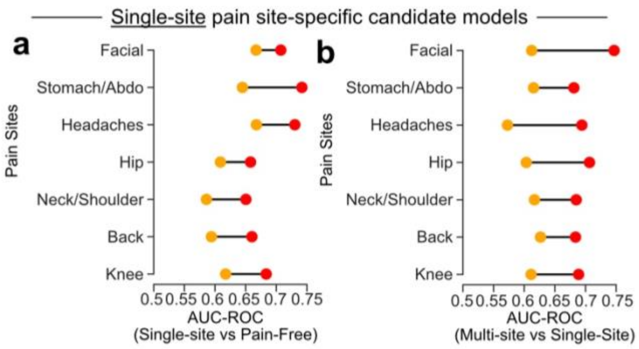

Supplementary Fig. 2 | Lack of specificity in single-site pain site-specific models. A total of 14 single-site acute and chronic pain models were derived in the discovery set and examined in the validation set to dissociate single-site risk factors from multi-site. AUC-ROC discrimination for each acute (orange) and chronic (red) pain site model comparing **a.** single-site to pain-free and **b.** multi-site to single-site. Area Under the Curve (AUC); Receiver Operating Characteristic Curve (ROC).

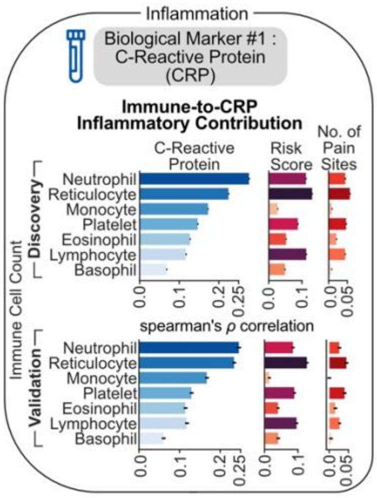

Supplementary Fig. 3 | Supplementary analysis of immune-inflammatory profile. Spearman's pho association (non-parametric estimation) between 7 different immune cell counts from a standard complete blood count and c-reactive protein, the inflammation marker examined, our risk score and the number of pain sites in both the discovery and validation sets. Error bars were estimated from 1,000 bootstrap samples.

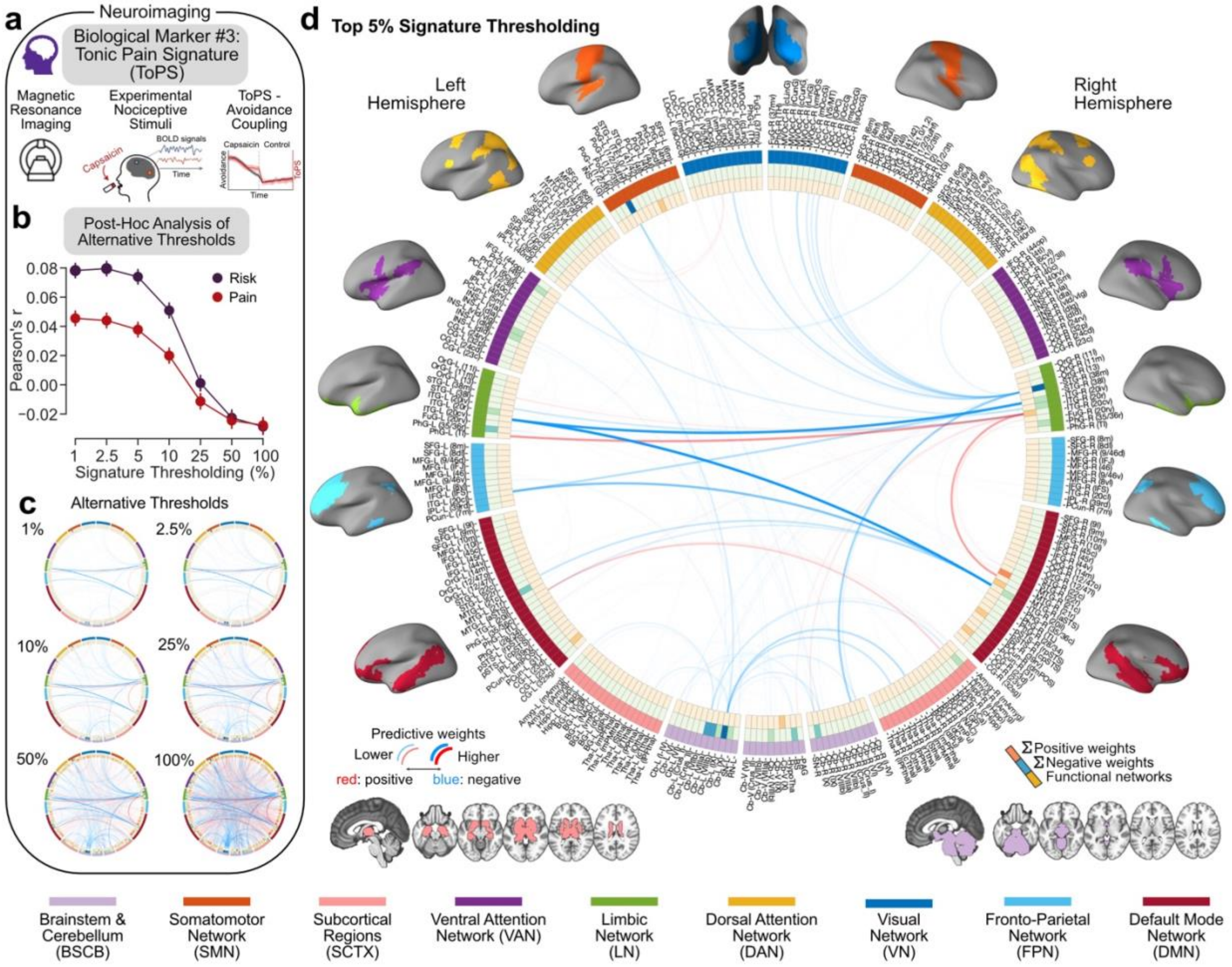

Supplementary Fig. 4 | Tonic Pain Signature (ToPS) neuroimaging marker. a. Tonic Pain Signature (ToPS) was used to capture blood oxygenation level dependant fluctuation following a capsaicin-induced sustained pain. Top 5% weights of the signature were used. Alternative thresholds and the top 5% thresholds shown in **b.** with their associations with the risk score and number of pain sites and **c.** with their respective connectivity patterns of alternative ToPS thresholds from the top 5% signature across each major networks**. d.** Connectivity pattern of the ToPS used in the study across each major brain networks. Each cell represents a brain region of interest colored based on the density of positive (in red) and negative (in blue) edges. Abbreviations: Brainstem and Cerebellum (BSCB); Somatosensory Network (SMN); Subcortex (SCTX); Ventral attention Network (VAN); Limbic Network (LN); Dorsal Attention Network (DAN); Visual Network (VN); Fronto-Parietal Network (FPN); Default Mode Network (DMN).

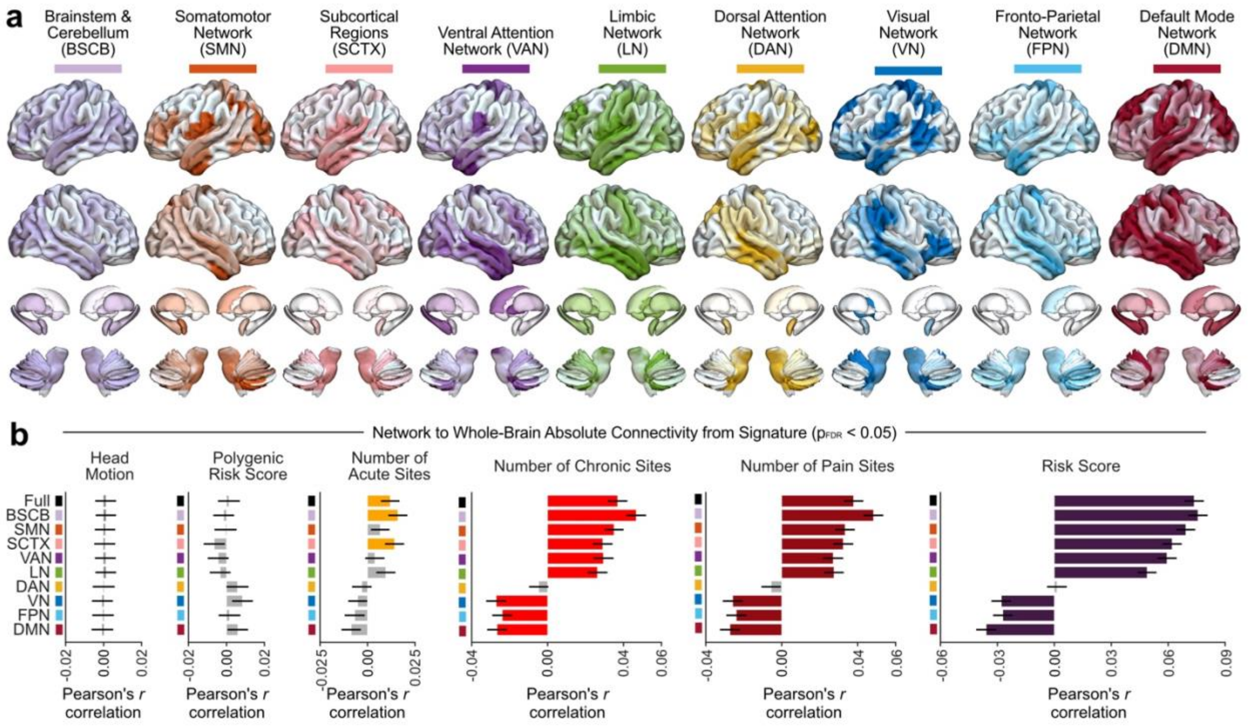

Supplementary Fig. 5 | Analysis of distinct network connectivity patterns from the ToPS. a. Visualization of the absolute connectivity network-to-whole brain expressed in the ToPS across each of the major networks. Network-to-whole brain connectivity measured using the sum of normalized dynamic conditional correlation connectivity across each parcel (max = 100). **b.** The association with head motion, our selected polygenic risk score, the number of acute, chronic, and combined pain sites as well as our risk score with each network. Error bars were estimated from 1,000 bootstrap samples. Significance was obtained from a two-tailed Pearson's *r* correlation and comparisons were FDR-corrected. Abbreviations: False Discovery Rate (FDR); Brainstem and Cerebellum (BSCB); Somatosensory Network (SMN); Subcortex (SCTX); Ventral attention Network (VAN); Limbic Network (LN); Dorsal Attention Network (DAN); Visual Network (VN); Fronto-Parietal Network (FPN); Default Mode Network (DMN)
