# Supplementary material for: A prognostic risk score for development and spread of chronic pain
Source: Nat Med. 2023 Jul 6;29(7):1821–31. doi: 10.1038/s41591-023-02430-4 (PMC10353938; doi:10.1038/s41591-023-02430-4)
Supplement: Supplementary file 2 — Reporting summary [file 41591_2023_2430_MOESM2_ESM.pdf]

Corresponding author(s): Christophe Tanguay-Sabourin and Etienne Vachon-Presseau

Last updated by author(s): Aug 22, 2022

Reporting Summary

Nature Portfolio wishes to improve the reproducibility of the work that we publish. This form provides structure for consistency and transparency in reporting. For further information on Nature Portfolio policies, see our Editorial Policies and the Editorial Policy Checklist.

Statistics

For all statistical analyses, confirm that the following items are present in the figure legend, table legend, main text, or Methods section.

n/a	Confirmed	
☐	☒	The exact sample size (*n*) for each experimental group/condition, given as a discrete number and unit of measurement
☐	☒	A statement on whether measurements were taken from distinct samples or whether the same sample was measured repeatedly
☐	☒	The statistical test(s) used AND whether they are one- or two-sided<br>*Only common tests should be described solely by name; describe more complex techniques in the Methods section.*
☐	☒	A description of all covariates tested
☐	☒	A description of any assumptions or corrections, such as tests of normality and adjustment for multiple comparisons
☐	☒	A full description of the statistical parameters including central tendency (e.g. means) or other basic estimates (e.g. regression coefficient) AND variation (e.g. standard deviation) or associated estimates of uncertainty (e.g. confidence intervals)
☐	☒	For null hypothesis testing, the test statistic (e.g. *F*, *t*, *r*) with confidence intervals, effect sizes, degrees of freedom and *P* value noted<br>*Give P values as exact values whenever suitable.*
☒	☐	For Bayesian analysis, information on the choice of priors and Markov chain Monte Carlo settings
☒	☐	For hierarchical and complex designs, identification of the appropriate level for tests and full reporting of outcomes
☐	☒	Estimates of effect sizes (e.g. Cohen's *d*, Pearson's *r*), indicating how they were calculated

Our web collection on statistics for biologists contains articles on many of the points above.

Software and code

Policy information about availability of computer code

Data collection	No software was used for data collection. Data was obtained from the UK Biobank. The acquisition and collection is public and presented in great details in previous protocol papers (Bycroft et al. 2018, Nature) or online (https://www.ukbiobank.ac.uk/learn-more-about-uk-biobank).
Data analysis	Python softwares (version) used: Numpy (1.22.0), Pandas (1.3.5), Sklearn (1.0.2), Nilearn (0.9.0) and Nltools (0.4.5). R software: Qgraph (1.9.2) and Matlab custom code: https://github.com/cocoanlab/tops. Manuscript analysis code are currently available (https://github.com/EVPlab). The codes are currently being cleaned to be more user friendly. The final version of the cleaned codes will be uploaded prior to the publication of the manuscript.

For manuscripts utilizing custom algorithms or software that are central to the research but not yet described in published literature, software must be made available to editors and reviewers. We strongly encourage code deposition in a community repository (e.g. GitHub). See the Nature Portfolio guidelines for submitting code & software for further information.

Data

Policy information about availability of data

All manuscripts must include a data availability statement. This statement should provide the following information, where applicable:

- Accession codes, unique identifiers, or web links for publicly available datasets
- A description of any restrictions on data availability
- For clinical datasets or third party data, please ensure that the statement adheres to our policy

All data are provided from the UK Biobank and are available to other investigators online upon permission granted by www.ukbiobank.ac.uk. Restrictions apply to the availability of these data, which were used under license for the current study (Project ID: 20802). The NFBC data are available upon request from the University of Oulu, Infrastructure for Population Studies (see; https://www.oulu.fi/en/university/faculties-and-units/faculty-medicine/northern-finland-birth-cohorts-and-arctic-biobank)s. Permission to use the data can be requested for research purposes via an electronic Material request portal (Greip). Prevent-AD data can be accessed openly at https://openpreventad.loris.ca while most of the other information, sensitive by nature, is accessible by qualified researchers at https://registeredpreventad.loris.ca.

Human research participants

Policy information about studies involving human research participants and Sex and Gender in Research.

Reporting on sex and gender	Sex of participant (as reported from the UK Biobank) was entered as a feature in our predictive models. We found an effect of sex on pain conditions, as previously reported in the literature, and we reported it along other demographics. No information regarding gender identity specifically was collected.
Population characteristics	UK Biobank is a large sample of participants recruited in the United Kingdom aged between 40-70 years old at baseline (51-55% female) with predominantly participants of white ethnicity (94-96%). Participant with chronic pain reported pain at any of the 8 body sites examined for more than 3 months.<br>The Northern Finland Birth Cohort (NFBC1966) was originally composed of 12,068 newborns in 1966. The data utilized for this study was obtained at 31- and 46-year follow-ups conducted in 1997-1998 and 2012-2014, respectively 45. Cross sectional analysis was conducted at the 46-year follow-up with a final population of 5,525 and only participants with complete data in the required pain questionnaires were included. A longitudinal analysis of participants present at both the 31-year and 46-year visit was also conducted with a total population of 4,710.<br>The Prevent-AD dataset is an observational cohort originally comprising 349 adults aged older than 60 years old at baseline visit (i.e., between 2011 and 2017) who met the eligibility criteria of investigation. Cross-sectional analysis was conducted on data available from a total of 178 individuals.
Recruitment	UK Biobank consist of 9.2M individuals that were invited by mail. About 500,000 participants provided informed consent and visited an assessment center during a baseline visit. The Northern Finland Birth Cohorts program (NFBC) was initiated in the 1960s in the two northernmost provinces of Finland to study risk factors involved in pre-term birth and intrauterine growth retardation, and the consequences of these early adverse events on subsequent morbidity and mortality. The NFBC1966 includes 12,068 live births to mothers in the two northern-most provinces of Finland representing 96.3% of births in the target region. The Prevent-AD dataset is an observational cohort that recruited healthy individuals at-risk of developing Alzheimer's disease (AD) due to a first-degree family of AD.
Ethics oversight	UK Biobank was approved by the National Information Governance Board for Health and Social Care and the National Health Service North West Multicenter Research Ethics Committee (reference number 06/MRE08/65). All participants gave written, informed consent, and the study was approved by the Research Ethics Committee (REC number 11/NW/0382). Further information on the consent procedure can be found elsewhere (https://biobank.ctsu.ox.ac.uk/crystal/field.cgi?id=200). Each follow-up study of the NFBC1966 has been evaluated by the regional ethical committee of the Norther Ostrobothnia Hospital District (EETTMK 94/11, 17.09.2012). The use of the NFBC data is based on cohort participants' written informed consent at their latest follow-up study. Participants in the Prevent-AD cohort provided written informed consent to participate at each follow-up visit including questionnaires and multimodal imaging assessments. Protocols, consent forms and study procedures were approved by McGill Institutional Review Board and/or Douglas Mental Health University Institute Research Ethics Board.

Note that full information on the approval of the study protocol must also be provided in the manuscript.

Field-specific reporting

Please select the one below that is the best fit for your research. If you are not sure, read the appropriate sections before making your selection.

☒ Life sciences ☐ Behavioural & social sciences ☐ Ecological, evolutionary & environmental sciences

For a reference copy of the document with all sections, see nature.com/documents/nr-reporting-summary-flat.pdf

Life sciences study design

All studies must disclose on these points even when the disclosure is negative.

Sample size	No sample-size calculation was done. A total of 493,211 participants were included from the UK Biobank, which is sufficient to train linear predictive models using 99 features. The models were then tested in two independent cohorts to avoid overfitting.
Data exclusions	Participants with more than 20% of missing data among the 99 features used or with missing data at any of the acute or chronic pain sites were excluded (< 2.5% exclusion). To ensure the findings of the study to be as generalizable as possible to the greater population, no other exclusion criteria were applied.
Replication	We divided the UK Biobank data in a training set and a testing set to validate our results. The model was derived on individual attending only the baseline visit (445,132) and validated in an out of sample set of participants attending a 6-10 years follow-up visit (48,079 participants).
Randomization	No randomization was used. Discovery and validation groups were determined according to participant's attendance to a follow-up visit.
Blinding	No blinding was performed. The machine-learning algorithm used cross validation and the results were tested in the out of sample participants.

Reporting for specific materials, systems and methods

We require information from authors about some types of materials, experimental systems and methods used in many studies. Here, indicate whether each material, system or method listed is relevant to your study. If you are not sure if a list item applies to your research, read the appropriate section before selecting a response.

Materials & experimental systems

n/a	Involved in the study	
☒	☐	Antibodies
☒	☐	Eukaryotic cell lines
☒	☐	Palaeontology and archaeology
☒	☐	Animals and other organisms
☒	☐	Clinical data
☒	☐	Dual use research of concern

Methods

n/a	Involved in the study	
☒	☐	ChIP-seq
☒	☐	Flow cytometry
☐	☒	MRI-based neuroimaging

Magnetic resonance imaging

Experimental design

Design type	UK Biobank brain imaging resting-state functional MRI scans
Design specifications	Single 6 minutes resting-state run, eyes open.
Behavioral performance measures	The number of self-reported pain sites, specific pain body sites and our derived risk score.

Acquisition

Imaging type(s)	UK Biobank brain imaging data: structural (T1 susceptibility-weighted) and resting-state functional scans.
Field strength	3T
Sequence & imaging parameters	The brain imaging protocols implemented in the UKBiobank are described in Miller et al., Nature Neuroscience 2016. In brief, The T1 structural protocol is acquired at 1mm isotropic resolution using a three-dimensional (3D) MPRAGE acquisition, with inversion and repetition times optimized for maximal contrast. Six minutes resting state resting-state fMRI used 2.4-mm spatial resolution and TR = 0.735 s, with multiband acceleration factor of 8.
Area of acquisition	Whole brain

Diffusion MRI ☐ Used ☒ Not used

Preprocessing

Preprocessing software	Minimal processing was done according to Miller et al., Nature Neuroscience 2016. Additional processing was conducted including despiking (AFNI from Nipype), 6-mm kernel smoothing (Nilearn), and resampling to 3-mm (for storage purposes) to

resemble an a-priori brain-based signature for sustained pain (ToPS; see Lee et al., 2021 Nature Medicine).

Normalization	Spatial normalization was done using non-linear registration based on the structural T1 images.
Normalization template	FSL's MNI152 and UK Biobank.
Noise and artifact removal	MRI-based covariates included head motion (linear, squared, and cubed), imaging site, position in the scanner, and coil position (Z, Y, Z respectively). Two different deconfounding framework were tested as described in the Methods.
Volume censoring	N/A

Statistical modeling & inference

Model type and settings	Signature response was obtained by computing a dot product between the DCC and the ToPS weights. Pearson's r correlation and Cohen's d (pooled standard deviation) between pain and pain-free participants.
Effect(s) tested	The associations between the apriori derived brain signature (ToPS) with our derived risk score and the number of pain sites.

Specify type of analysis: ☒ Whole brain ☐ ROI-based ☐ Both

Statistic type for inference (See Eklund et al. 2016)	This study used resting state functional connectivity methods.
Correction	Significance of group comparisons was determined using false discovery rate (q = 0.05).

Models & analysis

n/a	Involved in the study
☐	☒ Functional and/or effective connectivity
☒	☐ Graph analysis
☐	☒ Multivariate modeling or predictive analysis

Functional and/or effective connectivity	DCC was used for Dynamic Connectivity following the same protocol as the one used to derive the Tonic Pain Signature (see Lee et al., 2021 Nature Medicine).
Multivariate modeling and predictive analysis	Dot product was used to extract the ToPS signature response from the DCC connectivity and the weights of the ToPS signature.
